# Dichotomous metabolic networks govern human ILC2 proliferation and function

**DOI:** 10.1038/s41590-021-01043-8

**Published:** 2021-10-22

**Authors:** Laura Surace, Jean-Marc Doisne, Carys A. Croft, Anna Thaller, Pedro Escoll, Solenne Marie, Natalia Petrosemoli, Vincent Guillemot, Valerie Dardalhon, Davide Topazio, Antonia Cama, Carmen Buchrieser, Naomi Taylor, Ido Amit, Olimpia Musumeci, James P. Di Santo

**Affiliations:** 1 Innate Immunity Unit, Institut Pasteur, Inserm U1223, Paris, France; 2 Université de Paris, Sorbonne Paris Cité, Paris, France; 3 Biology of Intracellular Bacteria Unit, Institut Pasteur, CNRS UMR 3525, Paris, France; 4 Bioinformatics and Biostatistics Hub, Center of Bioinformatics, Biostatistics, and Integrative Biology, Institut Pasteur, Paris, France; 5 Institut de Génétique Moléculaire de Montpellier, University of Montpellier, CNRS, Montpellier, France; 6 Department of Otolaryngology, Hospital ‘Mazzini’, Teramo, Italy; 7 Department of Maxillofacial and Otolaryngology, Hospital ‘F. Renzetti’, Lanciano, Italy; 8 Department of Immunology, Weizmann Institute of Science, Rehovot, Israel; 9 Unit of Neurology and Neuromuscular Disorders, Department of Clinical and Experimental Medicine, University of Messina, Messina, Italy

**Keywords:** Innate lymphoid cells, Interleukins

## Abstract

Group 2 innate lymphoid cells (ILC2s) represent innate homologs of type 2 helper T cells (T_H_2) that participate in immune defense and tissue homeostasis through production of type 2 cytokines. While T lymphocytes metabolically adapt to microenvironmental changes, knowledge of human ILC2 metabolism is limited, and its key regulators are unknown. Here, we show that circulating ‘naive’ ILC2s have an unexpected metabolic profile with a higher level of oxidative phosphorylation (OXPHOS) than natural killer (NK) cells. Accordingly, ILC2s are severely reduced in individuals with mitochondrial disease (MD) and impaired OXPHOS. Metabolomic and nutrient receptor analysis revealed ILC2 uptake of amino acids to sustain OXPHOS at steady state. Following activation with interleukin-33 (IL-33), ILC2s became highly proliferative, relying on glycolysis and mammalian target of rapamycin (mTOR) to produce IL-13 while continuing to fuel OXPHOS with amino acids to maintain cellular fitness and proliferation. Our results suggest that proliferation and function are metabolically uncoupled in human ILC2s, offering new strategies to target ILC2s in disease settings.

## Abstract (web summary)

ILC2 metabolism has been largely unexplored. Di Santo and colleagues examine metabolic profiles from naive and cytokine-activated ILC2s and find that IL-33-triggered ILC2s rely on distinct metabolic pathways to sustain proliferation and function.

## Main

ILCs have important roles in systemic as well as local tissue immunity and are involved in early immune responsiveness and regenerative processes that restore homeostasis^1–3^. Five ILC subsets (ILC1, ILC2, ILC3, lymphoid tissue inducer cells (LTi) and NK cells) have been described both in mice and humans based on their transcription factor dependency and signature cytokine production^4–10^. ILC2s rely on the transcription factor GATA-3 for development and for regulation of the expression of type 2 cytokines, including IL-5, IL-9 and IL-13, and amphiregulin in response to alarmins, such as IL-33 (refs. ^11–15^). ILC2s reside mostly in barrier tissues; however, they can also enter the circulation and traffic between tissues^16–20^. In mice, two subsets have been identified: the tissue-resident natural ILC2s (nILC2s) and the inflammatory ILC2s (iILC2s), which are generated following the induction of type 2 inflammation in response to IL-25 and have the capacity to migrate^21–23^. Whether this dichotomy exists in humans is still a matter of debate. Human circulating ILC2s are described as CD45RA^+^ naive cells, which reside in a resting state and are then recruited into tissues to become activated^17,24^. Circulating and tissue-resident human ILC2s have heterogeneous phenotypes and functions^1,7,25^, but their key regulators remain poorly defined.

Mitochondria play a central role in cellular metabolism and are integral to a functional immune response^26^. Immunometabolism studies on T cells have defined specific metabolic programs (glycolysis and OXPHOS) following activation and differentiation^27–31^. Metabolite tracing revealed that activated T cells are preferentially glycolytic and take up glutamine to replenish tricarboxylic acid (TCA) cycle intermediates during cell proliferation and cytokine production^32^. By contrast, memory T cells exhibit a metabolic switch to fatty acid (FA) metabolism, mitochondria fusion and increased respiration^33–35^. However, single-cell metabolic flux analysis revisited this concept of a direct naive-to-activated T cell metabolic switch, showing that naive T cells are metabolically heterogeneous and explore ‘metabolic checkpoints’ before engaging into a specific program, which would then dictate downstream function^30^. While ILC2s have been reported to rely on nutrients other than glucose, including FA^36,37^ and arginine^38^, it is not known how different metabolic identities in ILC2s are generated following activation and linked to their biological roles. Here, we show that circulating naive human ILC2s reside in a highly active metabolic state relying on branched chain amino acids (BCAAs) and arginine to support mitochondrial OXPHOS. Following activation, ILC2s leverage their enhanced amino acid metabolism for rapid proliferation but engage glycolysis for effector cytokine production. The independent regulation of proliferation and effector function by dichotomous metabolic pathways opens avenues for manipulating ILC2s in disease settings.

**Fig. 1:**
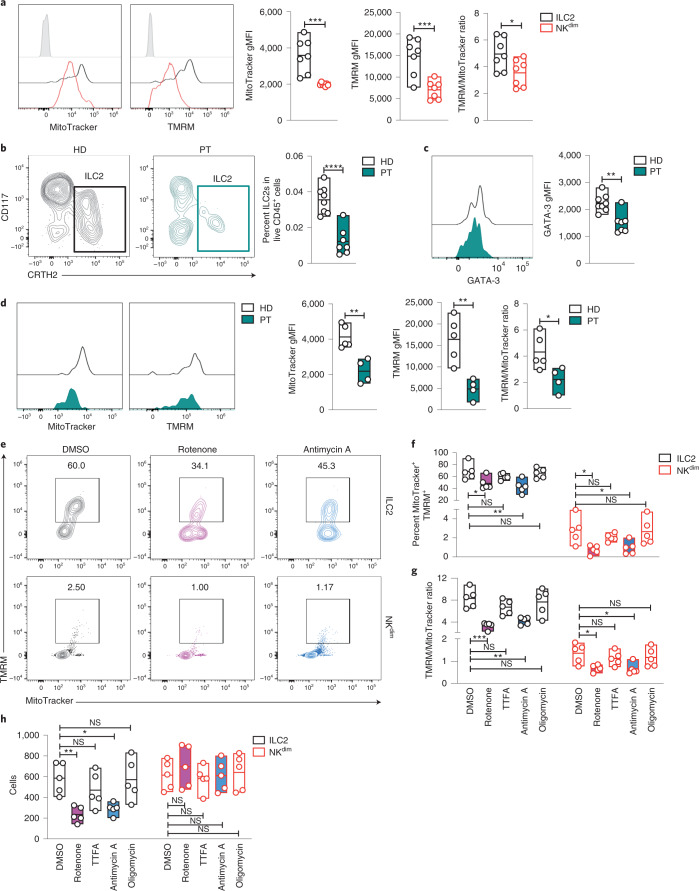
Complex I and III of the electron transport chain (ETC) support ILC2 survival. **a**, TMRM and MitoTracker measurement in freshly sorted ILC2s and NK^dim^ cells. Representative plots show MitoTracker and TMRM geometric mean fluorescence intensity (gMFI) and the ratio between the gMFI of TMRM and MitoTracker (*n* = 7). **b**–**d**, Comparison of healthy donors (HD; black) and individuals with MD (PT; green). Representative plots show the percentage of ILC2s in live CD45^+^ cells (**b**), GATA-3 gMFI in circulating ILC2s (**c**) (HD = 7; PT = 7) and MitoTracker and TMRM gMFI and the ratio between the geometric mean of TMRM and MitoTracker (**d**) (HD = 5; PT = 4). **e**–**h**, Freshly sorted ILC2s and NK^dim^ cells were cultured without additional cytokines for 18 h in DMSO, rotenone (1 µM), antimycin A (1 µM), 2-thenoyltriflouroacetone (TTFA; 1 µM) or oligomycin (1 µM). Representative TMRM and MitoTracker FACS plots (**e**), percentage of MitoTracker^+^TMRM^+^ cells in live CD45^+^ cells (**f**), the ratio between the geometric mean of TMRM and MitoTracker (**g**) and cell counts calculated by FACS (**h**) are shown (*n* = 5). The data in **a** are representative of five independent experiments with two to five donors each. Each dot in **b** represents one donor. The data in **c** and **d** are representative of two independent experiments with two to five healthy donors and two to four individuals with MD. The data in **e**–**h** are representative of three independent experiments with at least three donors each. Floating bars in **a**–**h** indicate the mean, minimum and maximum values within the dataset. Statistics were assessed by one-way analysis of variance (ANOVA) with a Dunnett correction; NS, not significant (*P* > 0.05); *P < 0.05; **P < 0.01; ***P < 0.001; ****P < 0.0001. Source data

**Extended Data Fig. 1:**
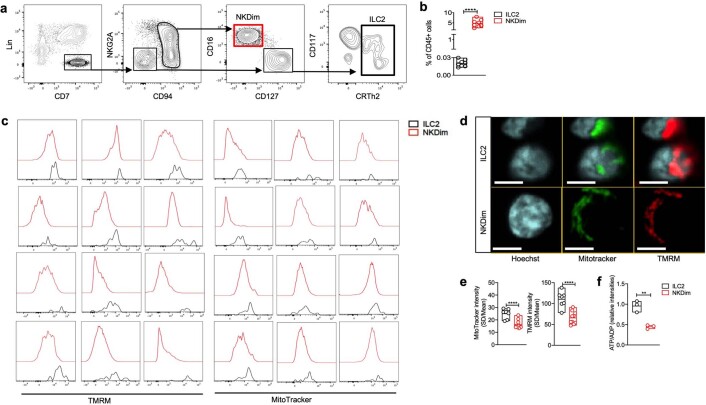
Human ILC2 have fused and polarized mitochondria. **a**, ILC2 Gating strategy Live CD45^+^ CD7^+^ Lin^-^ CD94^-^ NKG2A^-^ CD56^-^ CD16^-^ CD127^+^ CRTh2^+^ (ILC2) and Live CD45^+^ CD7^+^ Lin^-^ CD94^+^ CD16^+^ (NKDim). **b**, Percentage of ILC2 and NKDim in live CD45^+^ cells. **c**, FACS measurement of TMRM and MitoTracker in healthy donors of fresh ILC2 and NKDim (n = 12). **d**, Confocal microscopy in fresh ILC2 and NKDim (n = 7). **e**, MitoTracker and TMRM intensity (SD/Mean). **f**, ATP and ADP intensities quantified by label-free mass spectrometry in freshly sorted ILC2 and NKDim cells (n = 3). **a**-**b**, Data representative of 10 independent experiments with 2 to 4 donors each. **c**, Data from 12 healthy donors from at least 5 independent experiments. **d-e**, Data representative of 2 independent experiments with at least 3 donors. Dots are single cells in the analyzed field. Scale bar 5μm. **f**, Each dot represents a donor. A minimum of 2 technical replicates were analyzed per run. **b**, **e**, **f**, Floating bars indicate the mean, minimum and maximum values within the dataset. Statistics were assessed by two tailed t-test (**b,e,f**), not significant (ns), p > 0.05; **p < 0.01; ****p < 0.0001. Source data

**Extended Data Fig. 2:**
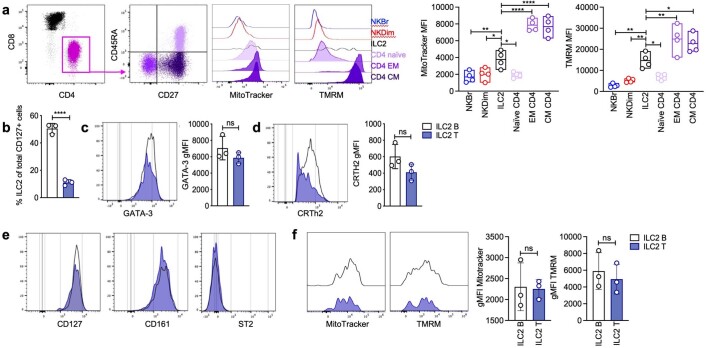
ILC2 have enhanced mitochondrial activity compared to naïve T cells. **a**, Gating strategy for CD4^+^ T cells subsets in the blood of healthy donors. FACS measurement of TMRM and MitoTracker gMFI in the indicated subsets (n = 4). **b**-**f**, Paired blood and tonsil samples were collected from pediatric donors. The percentage of ILC2 (**b**), GATA-3 gMFI (**c**), CRTH2 gMFI (**d**), representative plots of CD127, CD161, ST2 (**e**), TMRM and Mitotracker gMFI (**f**) were monitored by FACS (n = 3). **a**, Data representative of 2 experiments with at least 3 donors each. **b**-**f**, Data are representative of 2 independent experiments with 3 donors each. **a**, Floating bars indicate the mean, minimum and maximum values within the dataset. **b**-**f**, Bars are ± s.e.m. Statistics were assessed by one-way ANOVA with Dunnett correction (**a**) and two tailed t-test (**b-f**), not significant (ns), p > 0.05; *p < 0.05; **p < 0.01; ****p < 0.0001. Source data

We used MitoTracker Green FM and tetramethylrhodamine (TMRM) staining as described in ref. ^39^ to assess mitochondrial mass and membrane polarization (Δ*ψ*_m_) in human blood ILC2s isolated from healthy donors. CD56^+^CD16^+^ NK^dim^ cells (Extended Data Fig. 1a,b) were used as an ILC reference for comparison. Previous studies suggested that blood ILC2s were naive^17,24^; however, fluorescence-activated cell sorting (FACS) analysis revealed that they have two times the mitochondrial mass of NK^dim^ cells (Fig. 1a). Despite heterogeneity across donors (Extended Data Fig. 1c), TMRM intensity and TMRM/MitoTracker ratios were consistently higher in ILC2s at steady state (Fig. 1a). As mitochondria fission and fusion are associated with glycolysis and OXPHOS, respectively^40^, we studied mitochondrial morphology and confirmed that ILC2s have higher mitochondrial mass and polarization than that observed in NK cells and they present fused mitochondria (Extended Data Fig. 1d,e). Because of their relative scarcity in blood, we could not monitor real-time ILC2 oxygen consumption or glycolytic rates. Instead, we measured ATP and ADP by mass spectrometry. We found that circulating ILC2s have high cytosolic ATP/ADP ratios compared to that observed in NK^dim^ cells (Extended Data Fig. 1f and Supplementary Data 1), consistent with enhanced mitochondrial function and inhibition of glycolysis. Memory T cells have highly polarized mitochondria and enhanced OXPHOS compared to naive T cells^27–41^. When comparing TMRM and MitoTracker patterns in ILC2s versus in naive and central/effector memory CD4^+^ T cell subsets (Extended Data Fig. 2a), ILC2s showed a distinct mitochondrial signature, suggesting exposure to environmental signals.

We next assessed the bioenergetic profile of tissue ILC2s. Despite their scarcity (Extended Data Fig. 2b), tonsil ILC2s appeared to have similar phenotypic and functional profiles as their blood counterparts^27^. Tonsil ILC2s (detected as Lin^–^CD127^+^CD161^+^GATA-3^+^ST2^+^ cells; Extended Data Fig. 2c–e) showed similar mitochondria polarization and mass as blood ILC2s (Extended Data Fig. 2f), suggesting a close metabolic relationship. One explanation is that some tonsil ILC2s may be blood derived and not (yet) affected metabolically by local environmental signals, which could include IL-2, IL-7, IL-33 and/or other cytokines.

**Extended Data Fig. 3:**
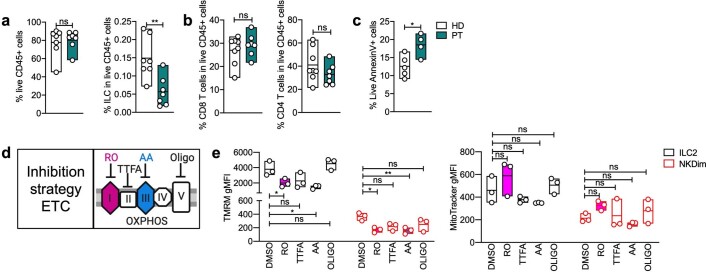
ILC2 are reduced in patients with mitochondrial diseases. **a**-**c**, Comparison of healthy donors (HD, black) and patients with mitochondrial diseases (PT, green). The percentage of live CD45^+^ cells, total ILC (**a**) CD8^+^ and CD4^+^ T cells (**b**) and live Annexin-V^+^ cells (**c**) was measured by FACS (HD = 7, PT = 7). **d**-**e**, Freshly sorted ILC2 and NKDim were cultured with no additional cytokines for 18h in DMSO, Rotenone (1uM), Antimycin A (1uM), TTFA (1uM) or Oligomycin (1uM), as indicated in the inhibition strategy (**d**). TMRM and MitoTracker gMFI were measured by FACS (**e**) (n = 3). **a-c**, Each dot represents a donor. **d-e**, Data representative of 3 independent experiments with at least 3 donors each. **a**-**c, e**, Floating bars indicate the mean, minimum and maximum values within the dataset. Statistics were assessed by two tailed t-test (**a**-**c**), and one-way ANOVA with Dunnett correction (**e**), not significant (ns), p > 0.05; *p < 0.05; **p < 0.01; ***p < 0.001; ****p < 0.0001. Source data

Individuals with MDs (impaired OXPHOS) have a twofold reduction in frequencies of blood NK^dim^ cells and a preferential loss of long-lived ‘memory-like’ NK^dim^ cells^39^. Reductions in blood ILC2s in individuals with MDs were even more profound (about 80%) (Fig. 1b and Extended Data Fig. 3a), while other lymphoid subsets (CD4^+^ and CD8^+^ T cells) were less affected (Extended Data Fig. 3b). Interestingly, residual ILC2s in individuals with MDs showed decreased GATA-3 expression (Fig. 1c), decreased mitochondrial mass and polarization (Fig. 1d) and more Annexin-V^+^ cells (Extended Data Fig. 3c), suggesting a requirement for mitochondrial function in ILC2 homeostasis.

As defects in components of the mitochondrial ETC underlie MDs in affected individuals (Supplementary Table 1), we corroborated these findings by exposing blood ILC2s to ETC inhibitors (Extended Data Fig. 3d,e). Inhibition of complexes I (rotenone) and III (antimycin A) significantly decreased the percentage of MitoTracker^+^TMRM^+^ cells (Fig. 1e,f) and impaired membrane potential, but not mitochondrial mass, in both ILC2s and NK^dim^ cells (Fig. 1g and Extended Data Fig. 3e). We observed that rotenone and antimycin A, but not complex II inhibition (TTFA), strongly impaired ILC2 survival, whereas NK^dim^ cells were largely unaffected (Fig. 1h). These results further support the essential role for active ETC complex I and complex III for survival of blood ILC2s.

**Fig. 2:**
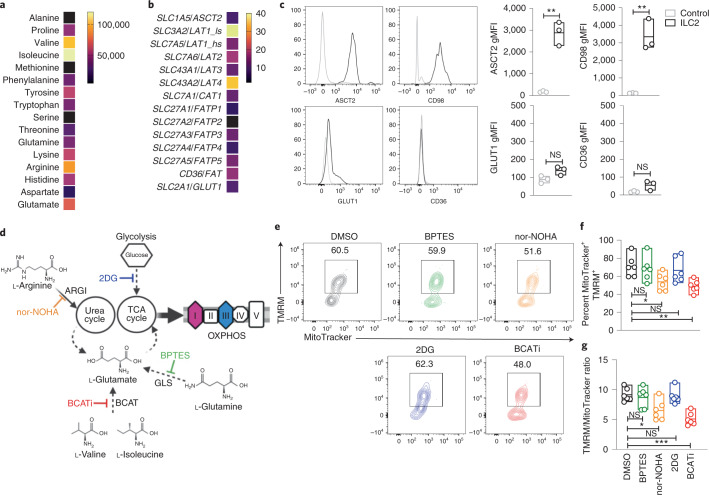
BCAA and arginine metabolism fuel OXPHOS in steady-state ILC2s. **a**, Heat map of amino acid intensities quantified by label-free mass spectrometry in freshly sorted ILC2s. **b**, Nutrient receptor analysis in fresh ILC2s by RNA sequencing (RNA-seq) (median value among three donors; relative expression). **c**, Representative plots of ASCT2 (glutamine transporter), CD98 (LAT1; large amino acid transporter), GLUT1 (glucose transporter) and CD36 (FA receptor) and MFI quantification of each receptor versus isotype control (*n* = 3). **d**–**g**, Freshly sorted ILC2s were cultured without additional cytokines for 18 h in DMSO, *N*^ω^-hydroxy-nor-arginine (nor-NOHA; 1 µM), BCAA transferase inhibitor (BCATi; 1 µM), bis-2-(5-phenylactamido-1,3,4-thiadiazol-2-yl)ethyl sulfide (BPTES; 1 µM) or 2-deoxy-d-glucose (2DG; 1 µM) to selectively inhibit the pathways depicted in the schematic in **d**; ARGI, arginase-1; GSL, glutaminase. A representative TMRM and MitoTracker FACS plot in ILC2s (**e**), the percentage of MitoTracker^+^TMRM^+^ cells (**f**) and the ratio between TMRM and MitoTracker MFI (**g**) (*n* = 6) are shown. The data in **a** are summarized from four donors, and a minimum of two technical replicates were analyzed per run. Data in **b** were extracted from an RNA-seq dataset, and the median was calculated from the values of three healthy donors. Data in **c** are representative of three independent experiments with at least three donors each. Data in **d**–**g** are summarized from two independent experiments with at least two donors, and plots are representative of a total of four independent experiments. The floating bars in **c**, **f** and **g** indicate the mean, minimum and maximum values within the dataset. Statistics were assessed by two-tailed *t*-test (**c**) and one-way ANOVA with Dunnett correction (**f** and **g**); NS, not significant (*P* > 0.05); *P < 0.05; **P < 0.01; ***P < 0.001. Source data

**Extended Data Fig. 4:**
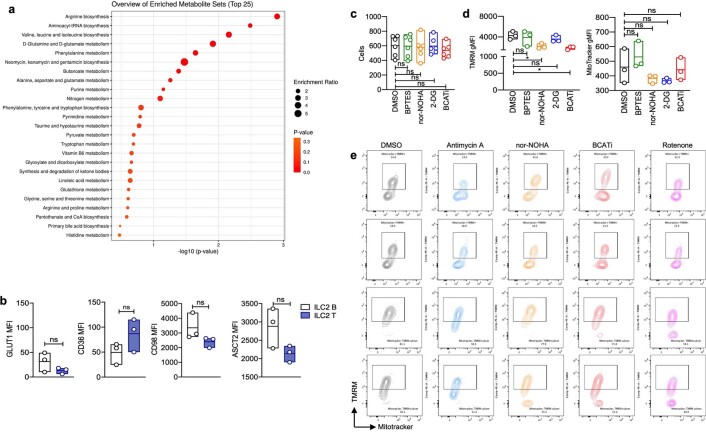
Branched amino acid and Arginine metabolism support OXPHOS in circulating ILC2. **a**, Enrichment analysis done by Metaboanalyst. Metabolite intensities were used as input and human metabolome was used as reference. **b**, Paired blood and tonsil samples were collected from pediatric donors. GLUT1, CD36, CD98 and ASCT2 were measured by FACS (n = 3). **c**-**d**, Freshly sorted ILC2 were cultured w/o additional cytokines for 18h in DMSO, nor-NOHA (1uM), BCATi (1uM), BPTES (1uM) or 2DG (1uM). Cell counts (**c**) (n = 6) and TMRM and MitoTracker MFI (**d**) (n = 3) were measured by FACS. **e**, FACS analysis of TMRM and Mitotracker staining in ILC2 cultured w/o additional cytokines for 18h in DMSO, Rotenone (1uM), Antimycin A (1uM), nor-NOHA (1uM) and BCATi (1uM). **a**, Data summarized from 4 donors. A minimum of 2 technical replicates were analyzed per run. **b**, Data are representative of 2 independent experiments with 3 donors each. **c**-**d**, Data are representative of 4 independent experiments with at least 2 donors each. **e**, Data are shown for 4 donors from 2 independent experiments. **b**-**d**, Floating bars indicate the mean, minimum and maximum values within the dataset. Statistics were assessed by two tailed t-test (**b**) and one-way ANOVA with Dunnett correction (**c**-**d**), not significant (ns), p > 0.05; *p < 0.05. Source data

We next purified ILC2s and analyzed the cellular metabolome using high-resolution mass spectrometry (Supplementary Data 1). Pathway analysis revealed an enrichment in metabolites from arginine, BCAAs (valine, leucine/isoleucine) and glutamine metabolism (Extended Data Fig. 4a). Valine and isoleucine were the most abundant amino acids in circulating ILC2s, followed by arginine and glutamate (Fig. 2a). Arginine and glutamine/glutamate have been described to play a role in lymphocyte proliferation^38,41,42^, while BCAAs are essential amino acids required for mTOR activation^43^. We detected enhanced expression of *SLC3A2* (light subunit of the LAT1 receptor) and *SLC43A2* (LAT4) in ILC2s (Fig. 2b). LAT1 and LAT4 preferentially transport BCAAs. Steady-state blood ILC2s showed high surface expression of CD98 (LAT1) but not GLUT1 (glucose transporter) or CD36 (FA transporter). ASCT2 (glutamine transporter) was also highly expressed (Fig. 2c), providing an explanation for the high glutamate levels observed in circulating ILC2s (Fig. 2c). We found a similar pattern of nutrient transporter expression on tonsil ILC2s (Extended Data Fig. 4b).

**Extended Data Fig. 5:**
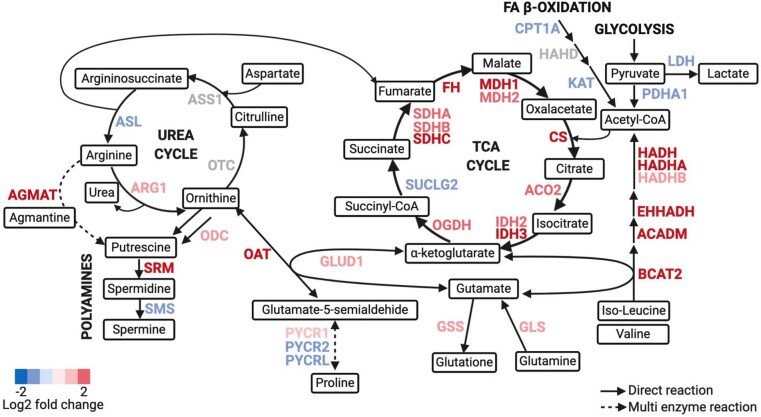
Branched amino acid and Arginine are used as building blocks to sustain TCA cycle and OXPHOS. Enzyme analysis comparing RNA-seq data to published NK cell dataset for the calculation of the fold change. Data extracted from RNA-seq dataset. Median calculated from values of 3 healthy donors. Source data

Pharmacological inhibition of glycolysis (2DG), arginase-1 (nor-NOHA), glutamine conversion into glutamate (BPTES) and BCAA transaminase that converts BCAA into glutamate (BCATi) (Fig. 2d) did not impact ILC2 cell number at the concentration used (Extended Data Fig. 4c). However, inhibition of arginase-1 and BCAT resulted in a reduced percentage of MitoTracker^+^TMRM^+^ ILC2s (Fig. 2e,f), with a significant reduction in mitochondrial Δ*ψ*_m_ (Extended Data Fig. 4d). The TMRM-to-MitoTracker ratio was also decreased (Fig. 2g), suggesting an impact on OXPHOS more than mitochondria remodeling or biogenesis. Given the observed heterogeneity in freshly sorted ILC2s (Extended Data Fig. 1c), we analyzed individual ILC2 TMRM profiles after treatment with OXPHOS inhibitors. Responses were uniform (Extended Data Fig. 4e), suggesting that the dependence of circulating ILC2s on OXPHOS/oxygen consumption and amino acids represents a fundamental property required to sustain mitochondrial activity. Corroborating these findings, we found upregulation of enzymes involved in the TCA cycle but low expression of enzymes involved in glycolysis and FA oxidation (Extended Data Fig. 5 and Supplementary Data 2). We also observed upregulation of enzymes involved in the conversion of BCAAs into glutamate and acetyl-CoA as well as upregulation of arginase-1 and ornithine aminotransferase, consistent with the role of BCAAs and arginine in fueling the TCA cycle (Extended Data Fig. 5) also described in differentiated T cells^44^. Together, our data show that circulating ILC2s are not in a resting metabolic state but already show elevated profiles characterized by increased BCAA and arginine levels that sustain OXPHOS required for cell fitness and survival.

**Fig. 3:**
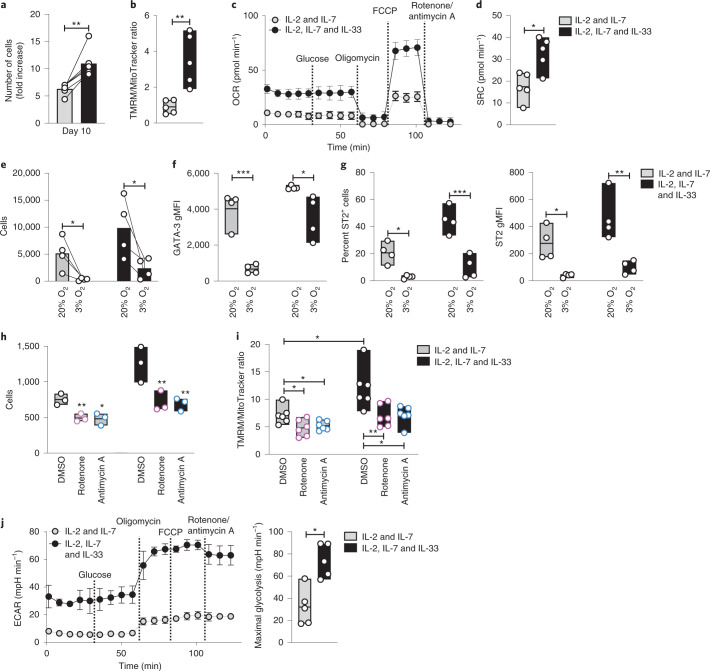
Activated ILC2s increase oxygen consumption and glycolytic capacity. **a**–**d** and **h**–**j**, ILC2s were expanded for 7 d in the presence of IL-2 and IL-7, and then IL-33 was added or not for 72 h. **a**, Number of cells at day 10 (*n* = 6). **b**, Ratio between TMRM and MitoTracker MFI (*n* = 5). **c**,**d**, Seahorse measurements following the addition of glucose, oligomycin, fluoro-carbonyl cyanide phenylhydrazone (FCCP) and a combination of rotenone and antimycin A, including OCR (**c**) and SRC (maximal respiratory capacity after FCCP – basal respiratory capacity) (**d**) (*n* = 5). **e**–**g**, Cells were cultured at 20% or 3% oxygen in IL-2 and IL-7 or IL-2, IL-7 and IL-33 for 5 d. Cell count (**e**), GATA-3 expression (**f**), percent ST2^+^ cells and ST2 expression (**g**) were analyzed by FACS. **h**,**i**, Cells were treated for the last 18 h with 1 µM rotenone, 1 µM antimycin A or DMSO (*n* = 5). The total cell counts (**h**) (*n* = 3) and the ratio between TMRM and MitoTracker MFI (**i**) (*n* = 6) were calculated by FACS. **j**, Extracellular acidification rate (ECAR) and maximal glycolysis were analyzed by Seahorse, performed as in **c** and **d** (*n* = 5). Data in **a**–**d** and **j** are representative of five independent experiments with three to five donors each. Data in **e**–**i** are representative of three independent experiments with at least three donors each. The bars in **a**, **c**, **e** and **j** (left plot) represent mean ± s.e.m., and the floating bars in **b**, **d**, **f**–**i** and **j** (right plot) indicate the mean, minimum and maximum values within the dataset. Statistics were assessed by two-tailed *t*-test (**a**–**d** and **j**) and one-way ANOVA with Tukey correction (**e**–**i**); NS, not significant (*P* > 0.05); *P < 0.05; **P < 0.01; ***P < 0.001. Source data

**Extended Data Fig. 6:**
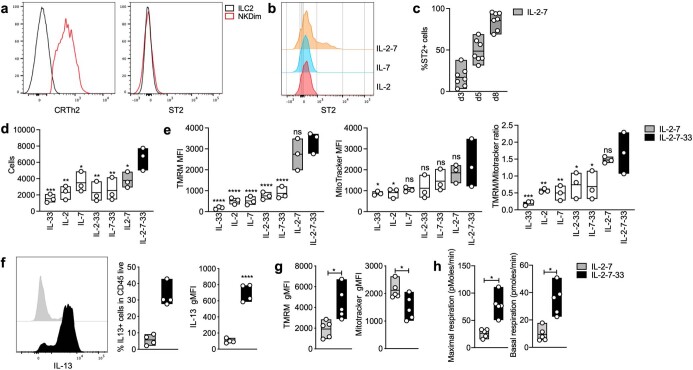
Activated ILC2 upregulate ST2 and enhance OXPHOS. **a**, Representative FACS plots of CRTh2 and ST2 in blood ILC2. **b**, ILC2 were cultured for 18h with IL-2, IL-7 or IL-2-7 and levels of ST2 were monitored by FACS. **c**, ILC2 were expanded for 3, 5 or 7 days in IL-2-7 and ST2 was measured by FACS (n = 7). **d,e**, ILC2 were expanded for 7 days in the indicated cytokine combinations. Cell number was assessed by trypan blue (**d**), TMRM and MitoTracker gMFI were measured by FACS (**e**) (n = 3). **f**-**h**, ILC2 were expanded for 7 days in IL-2-7 and then IL-33 was added or not for 72h. Levels of IL-13 (**f**) (n = 4), TMRM and MitoTracker (**g**) were measured at d10 by FACS (n = 5). **h**, Maximal and basal respiration were measured by seahorse upon addition of Glucose, Oligomycin, FCCP and a combination of Rotenone + Antimycin A (n = 5). **a**,**b** Plot representative of 3 different donors. **c-e**, Data are representative of two experiments with 3 to 4 donors each. **f**, Data are representative of 3 experiments with at least 3 donors each. **g-h**, Data are representative of 5 independent experiments with 3 to 5 donors each. **c**-**h**, Floating bars indicate the mean, minimum and maximum values within the dataset. Statistics were assessed by one-way ANOVA with Tukey correction (**d,e**) and two tailed t-test (**f-h**), not significant (ns), p > 0.05; *p < 0.05; **p < 0.01; ***p < 0.001; ****p < 0.0001. Source data

To gain insight into the metabolic changes that circulating ILC2s undergo following exposure to tissue-derived cytokines, we cultured sorted blood ILC2s with IL-2 and IL-7, which support basal proliferation without activation^11,45^, or with IL-2, IL-7 and IL-33 that can fully activate ILC2s with robust proliferation and secretion of type 2 cytokines^11,45,46^. Circulating ILC2s are CRTH2^+^ST2^–^ (Extended Data Fig. 6a), and a combination of IL-2 and IL-7 upregulated the IL-33 receptor (ST2) (Extended Data Fig. 6b,c), allowing IL-33 to exert its effects. Accordingly, IL-33 alone (or the combinations of IL-2 and IL-33 or IL-7 and IL-33) did not promote ILC2 proliferation or mitochondria polarization (Extended Data Fig. 6d,e). As expected, basal proliferation in cells treated with IL-2 and IL-7 was enhanced by IL-33 (Fig. 3a), and production of type 2 cytokines was clearly induced (Extended Data Fig. 6f). ILC2s stimulated with IL-2, IL-7 and IL-33 showed an increase in mitochondrial Δ*ψ*_m_ and a slight decrease in mitochondrial mass (Fig. 3b and Extended Data Fig. 6g). Analysis of oxygen consumption rate (OCR) showed that IL-33 increased ILC2 basal and maximal respiration (Fig. 3c and Extended Data Fig. 6h) as well as the spare respiratory capacity (SRC) compared to cells stimulated in IL-2 and IL-7 (Fig. 3d). Together, these results indicate that IL-33-activated ILC2s show enhanced OXPHOS and maintain high cellular fitness despite stresses associated with elevated metabolic activity.

**Extended Data Fig. 7:**
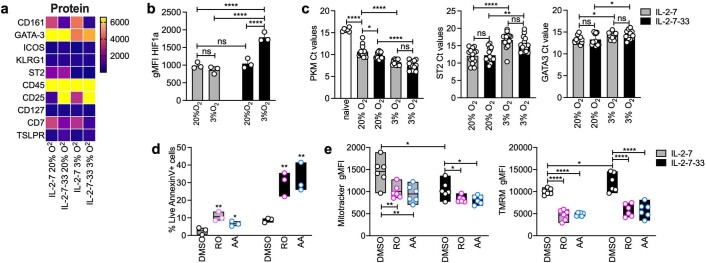
Oxygen consumption is crucial upon ILC2 activation. **a**-**c**, Cells were cultured at 20% or 3% oxygen in IL-2-7 or IL-2-7-33 for 5 days. The gMFI of the indicated makers in the heatmap (**a**) and HIF1a gMFI (**b**) were analyzed by FACS (n = 3). The level of expression of PKM, ST2 and GATA-3 transcripts was measured by Biomark (**c**) (n = 15). **d**-**e**, 1uM RO, or 1uM AA or with DMSO were added for 18h to the culture. Annexin V and L/D (**d**) (n = 3), Mitotracker and TMRM MFI (**e**) (n = 6) were analyzed by FACS. **a**, Data pooled from 3 independent experiments with 3 donors each. **b**, Data representative of 3 independent experiments. **c**, Data pooled from 2 independent experiments. **d-e**, Data are representative of a total of 4 independent experiments with 3 donors each. **b**-**c**, Bars are ± s.e.m.; **d**-**e**, Floating bars indicate the mean, minimum and maximum values within the dataset. Statistics were assessed by one-way ANOVA with Tukey correction (**b-e**), not significant (ns), p > 0.05; *p < 0.05; **p < 0.01; ***p < 0.001; ****p < 0.0001. Source data

We next cultured ILC2s with or without IL-33 under hypoxic conditions (3% oxygen). We observed that hypoxia led to a decrease in ILC2 survival as well as a loss of GATA-3 and ST2 protein (both gMFI and percentage of positive cells; Fig. 3e–g and Extended Data Fig. 7a). We did not find significant changes in the expression of other ILC2-related proteins or more general lymphoid markers (Extended Data Fig. 7a), suggesting that hypoxia regulates a specific program centered around GATA-3 and ST2. As previously reported, the HIF-1α–PKM axis modulates murine ILC2 bioenergetic balance and IL-33 responsiveness^47^. We found enhanced expression of HIF-1α (Extended Data Fig. 7b) and PKM concomitant with a reduction in GATA-3 and ST2 transcription (Extended Data Fig. 7c) in IL-33-stimulated ILC2s under hypoxic conditions, confirming this HIF-1α–PKM–ST2 axis in human ILC2s. We further observed that inhibition of ETC complexes I and III impaired survival and accentuated apoptosis of cytokine-activated ILC2s (Fig. 3h and Extended Data Fig. 7d), resulting in a loss of mitochondrial membrane potential and mass (Fig. 3i and Extended Data Fig. 7e). Together, these results confirm the crucial role of ETC function and oxygen in the homeostasis of cytokine-activated ILC2s.

**Fig. 4:**
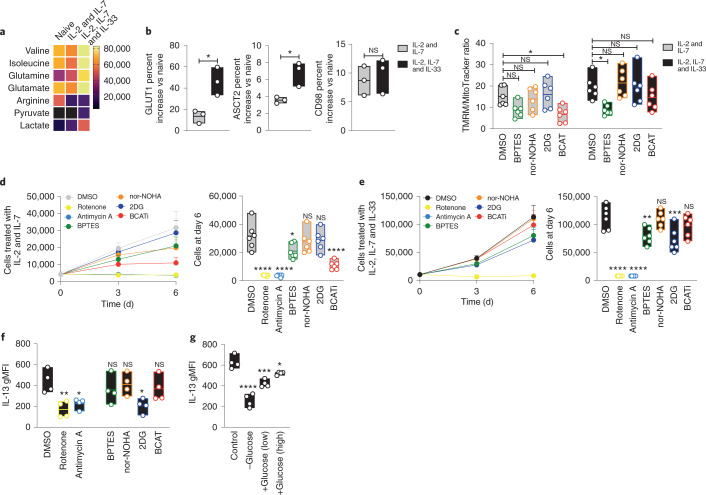
IL-33-stimulated ILC2s use amino acid metabolism to sustain cellular fitness and glucose for cytokine production. **a**–**c**, ILC2s were expanded for 7 d in IL-2 and IL-7, and IL-33 was added or not for 72 h. **a**, A heat map of amino acid intensities quantified by label-free mass spectrometry in naive cells and in ILC2s treated with either IL-2 and IL-7 or IL-2, IL-7 and IL-33 is shown. **b**, Percentage increase of nutrient receptors in primed (IL-2 and IL-7) or activated (IL-2, IL-7 and IL-33) cells versus in naive ILC2s (*n* = 3). **c**, ILC2s were cultured for 18 h with 1 µM BPTES, 1 µM 2DG, 1 µM nor-NOHA, 1 µM BCATi or DMSO in the indicated cytokine combinations. TMRM and MitoTracker were measured by FACS (*n* = 6). **d**,**e**, Cells were cultured for 6 d with IL-2 and IL-7 (**d**) or with IL-2, IL-7 and IL-33 (**e**) and 1 µM rotenone, 1 µM antimycin A, 1 µM BPTES, 1 µM 2DG, 1 µM nor-NOHA, 1 µM BCATi or DMSO. The cell counts at day 6 are shown (*n* = 6). **f**, Expression of IL-13. ILC2s were expanded in IL-2 and IL-7 for 7 d with or without inhibitors as described in **d** and **e**, and IL-33 was added for the last 6 h. **g**, ILC2s were expanded in IL-2 and IL-7 for 7 d, and IL-33 was added for the last 6 h to cells starved for 1 h and under a glucose add-back condition. IL-13 expression was monitored by FACS (**f**,**g**) (*n* = 4). Data in **a** are summarized from four donors, and a minimum of two technical replicates were analyzed per run. Data in **b** are representative of three independent experiments with three donors each. Data in **c** are summarized from two experiments with three donors each and are representative of a total of four independent experiments. Data in **d** and **e** are summarized from two experiments with three donors each and are representative of a total of three independent experiments. Data in **f** and **g** are representative of a total of three independent experiments with at least three donors. Floating bars in **b**–**g** indicate the mean, minimum and maximum values within the dataset. Bars in **d** and **e** (left plots) represent mean ± s.e.m. Statistics were assessed by two-tailed *t*-test (**b**) and one-way ANOVA (**c**–**g**) with Dunnett correction; NS, not significant (*P* > 0.05); *P < 0.05; **P < 0.01; ***P < 0.001. Source data

IL-33 stimulated ILC2s to increase their glycolytic capacity (Fig. 3j) while maintaining elevated OXPHOS and SRC (Fig. 3c,d). We hypothesized that ILC2s should rely on different nutrient sources to support their bioenergetic settings. Comparison of ILC2 metabolomes after cytokine stimulation showed elevated BCAAs and glutamine and a loss of arginine compared to naive ILC2s (Fig. 4a and Supplementary Data 1). Specific changes that accompanied IL-33 exposure included an increase in pyruvate and lactate, consistent with augmented glycolysis (Fig. 3j). IL-33-activated ILC2s showed an increase in the expression of GLUT1, ASCT1 and ASCT2 compared to naive levels (Fig. 4b), whereas CD98 levels were unchanged, suggesting that amino acid accumulation might not depend on increased uptake from the environment.

**Extended Data Fig. 8:**
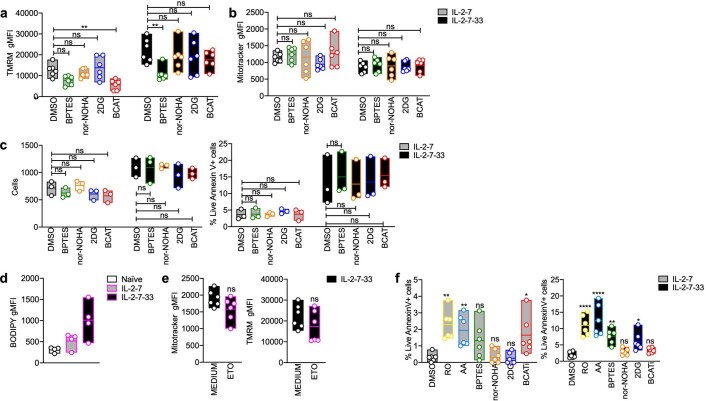
Activated ILC2 use different nutrients to sustain proliferation. **a**-**e**, ILC2 were expanded for 7 days in IL-2-7 and then IL-33 was added or not for 72h. Inhibitors or DMSO were added at the concentrations indicated in Fig. 4 for 18h. MitoTracker and TMRM (**a**-**b**) (n = 6), cell counts and AnnexinV (**c**) were monitored by FACS (n = 3). **d**-**e**, Bodipy (**d**) (n = 4), MitoTracker and TMRM (**e**) (n = 6) were analyzed by FACS in naïve, IL-2-7 and IL-2-7-33 stimulated ILC2. **f**, Cells were cultured for 6 days in IL-2-7 and 1uM RO, 1uM AA, 1uM BPTES, or 1uM 2DG, or 1uM nor-NOHA, or 1uM BCATi or DMSO. Annexin V was measured by FACS (n = 6). **a**-**b** Data are summarizing 2 experiments with 3 donors each. Representative of a total of 4 independent experiments. **c**-**e**, Data are representative of 3 independent experiments with at least 3 donors each. **f**, Data are summarizing 2 experiments with 3 donors each. Representative of a total of 3 independent experiments. Floating bars indicate the mean, minimum and maximum values within the dataset. Statistics were assessed by one-way ANOVA with Dunnett correction (**a**-**d**, **f**) and two tailed t-test in (**e**), not significant (ns), p > 0.05; *p < 0.05; **p < 0.01; ***p < 0.001; ****p < 0.0001. Source data

We next studied how these different nutrients are required for maintenance of mitochondrial activity in cytokine-activated ILC2s. We found that BCAAs are the main nutrient source maintaining OXPHOS in IL-2- and IL-7-stimulated ILC2s, while glutamine was also crucial in the context of IL-33 (Fig. 4c and Extended Data Fig. 8a). By contrast, BCATi did not have any obvious effect (in line with previous findings^48^), and no changes in ILC2 mitochondrial mass or viability were detected under any conditions (Extended Data Fig. 8b,c). We found an increase in the uptake of fluorescently labeled long-chain FA BODIPY only following IL-33 treatment (Extended Data Fig. 8d), consistent with a recent study showing the formation of FA lipid droplets following IL-33 chronic stimulation^35^. Still, inhibiting FA β-oxidation did not affect ILC2 mitochondrial mass or Δ*ψ*_m_ (Extended Data Fig. 8e), suggesting that FAs might not play a crucial role in sustaining ILC2 bioenergetic needs. Additional studies are needed to fully clarify the role of FAs in human ILC2s. Curiously, inhibition of glycolysis (using 2DG) did not impact mitochondrial polarization in cytokine-activated ILC2s, suggesting that these cells might couple glucose consumption to other functions.

To assess the effects of long-term inhibition of nutrient pathways, we extended the ILC2 cultures for 7 d (Fig. 4d,e and Extended Data Fig. 8f). As expected, cells did not survive in the presence of rotenone and antimycin A irrespective of cytokine combination. Long-term BPTES treatment impacted the survival of ILC2s when exposed to IL-2 and IL-7 or to IL-2, IL-7 and IL-33, while treatment with BCATi only had an effect in the absence of IL-33, consistent with our results on mitochondrial activity. In agreement with previous reports^49–52^ and our data on glycolytic capacity (Fig. 3j), long-term inhibition of glycolysis affected highly proliferative IL-33-stimulated ILC2s but not basal proliferation induced by IL-2 and IL-7 alone. These results indicate that amino acid metabolism remains a central orchestrator of cellular fitness during cytokine-induced ILC2 proliferation.

**Extended Data Fig. 9:**
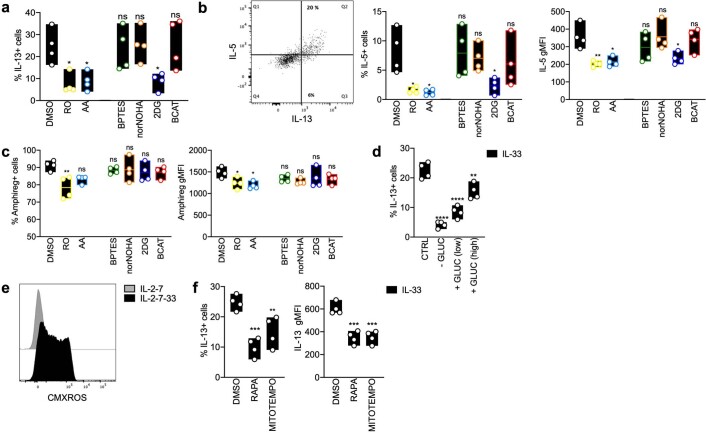
Activated ILC2 use different pathways to sustain function and proliferation. **a-c**, ILC2 were expanded in IL-2-7 for 7 days ± inhibitors as described in (Fig. 4d-e). IL-33 was added for the last 6 hours. Percentage of IL-13^+^ cells (**a**) IL-5+ cells and gMFI (**b**) and amphiregulin+ cells and gMFI (**c**) were measured by FACS (n = 4). **d**, ILC2 were expanded in IL-2-7 for 7 days and then IL-33 was added for the last 6 hours on cells starved for 1 hour and on glucose add-back condition. Percentage of IL-13^+^ cells was measured by FACS (n = 4). **e**, ILC2 were expanded for 7 days in IL-2-7 and then IL-33 was added or not for 72h. Levels of ROS was measured by FACS (n = 4). **f**, ILC2 were expanded in IL-2-7 for 7 days in the presence or not of the inhibitors MitoTempo (1uM) and Rapamycin (25nM). IL-33 was added for 6 hours. Percentage of IL-13^+^ cells was measure by FACS (n = 4). **a-d**, Data are representative of 3 independent experiments with at least 3 donors each. **e-f**, Data are representative of 2 independent experiments with at least 3 donors each. Floating bars indicate the mean, minimum and maximum values within the dataset. Statistics were assessed by one-way ANOVA with Dunnett correction (**a**-**f**), not significant (ns), p > 0.05; *p < 0.05; **p < 0.01; ***p < 0.001; ****p < 0.0001. Source data

We next studied the effect of metabolic inhibitors on ILC2 cytokine production. Blocking glycolysis or ETC complexes I or III decreased IL-13 and IL-5 production (Fig. 4f and Extended Data Fig. 9a,b). Amphiregulin production was less affected (Extended Data Fig. 9c), suggesting that pro- and anti-inflammatory ILC2 pathways might rely on different metabolic programs. Moreover, glucose starvation ablated IL-13 production, while glucose ‘add-back’ (at low or high concentration) partially restored IL-13 production (Fig. 4g and Extended Data Fig. 9d). It has been shown that glucose impacts lymphocyte function and proliferation through mTOR, an essential nutrient sensor^35^. Moreover, reactive oxygen species (ROS) have been reported to play a crucial role in mouse ILC activation^53^. We found that IL-33 induced ROS production in human ILC2s (Extended Data Fig. 9e). Treatment of ILC2s with a ROS scavenger or rapamycin (an mTOR inhibitor) reduced IL-13 production (Extended Data Fig. 9f). Taken together, these results demonstrate the unique role for glycolysis and ROS in coupling IL-33 activation to ILC2 cytokine production.

Here, we provide an in-depth characterization of human ILC2 metabolism at steady state and after cytokine activation. Our analysis identified the enhanced metabolic state of ILC2s compared to other innate lymphoid cell subsets and further deciphered the dichotomous nutrient pathways that sustain ILC2 survival, proliferation and function. Human ILC2 metabolism is poorly understood, with circulating ILC2s being described as resting naive cells with a migratory phenotype^17,24^. We show that they are instead highly energetic with a defined metabolic profile characterized by high expression of CD98 and elevated rates of amino acid uptake to sustain OXPHOS. These metabolic profiles provide cells with the capacity to respond to increased metabolic demands following activation^28,31^ and represent environment-specific metabolic adaptation^54,55^, suggesting that human circulating ILC2s might have metabolic ‘experience’. The heightened metabolic profile of circulating ILC2s was strongly OXPHOS/oxygen-dependent, as shown by the analysis of individuals with MDs and hypoxic experiments. Following activation, ILC2s do not undergo a metabolic switch from OXPHOS to glycolysis but rather maintain a dichotomous cellular metabolism with persistent OXPHOS and enhanced glycolysis. The former assures ILC2 survival (with glutamine as an anaplerotic substrate to maintain cell fitness), while glucose uptake and glycolysis sustain IL-13 production.

Our findings provide a working model for understanding how ILC2 metabolism conditions ILC function. Moreover, the peculiar metabolic features of steady-state and cytokine-activated ILC2s might represent potential targets for modulating these cells in diverse disease contexts.

## Methods

### Cell isolation from blood and tissue samples

Blood samples from healthy donors were randomly selected (age and sex) and obtained from Establissement Francais du Sang (EFS) under protocol HS 2105-24405. Peripheral blood mononuclear cells (PBMCs) from individuals with MD were obtained from the Unit of Neurology and Neuromuscular Disorders at ‘University of Messina’ with informed consent through an Institutional Review Board protocol (protocol 88/17 del 31 sett 2017) in compliance with ethical regulations. No statistical methods were used to predetermine sample sizes, but our sample sizes are similar to those reported in previous publications^9,11,17^. The number of participants for each experiment was dependent on donors’ availability. No direct compensation was given by the authors to the healthy donors or individuals with MD. Isolation of human PBMCs and single-cell suspensions was achieved by Ficoll-Paque (GE Healthcare) density gradient centrifugation. Data collection and analysis were not blinded to the conditions of the experiments.

### Cell culture and reagents

All in vitro culture experiments were performed in Yssel’s medium prepared in house by using IMDM (Invitrogen) and 0.25% (wt/vol) bovine serum albumin (Sigma), 1.8 mg liter^–1^ 2-amino ethanol, 40 mg liter^–1^ apo-transferrin, 5 mg liter^–1^ insulin and penicillin/streptomycin and supplemented with 2% human AB serum (EFS). FACS-sorted cells were plated in the presence of the human cytokines IL-2 (50 ng ml^–1^; Miltenyi), IL-7 (50 ng ml^–1^; Miltenyi) and IL-33 (50 ng ml^–1^; R&D), which were provided in various combinations and at specific times as indicated. For the hypoxia experiments, cells were cultured at 3% oxygen in the XVivo System (BioSperix) at constant pressure, humidity and 5% CO_2_. Chemical inhibitors used to study metabolic pathways (2DG, BPTES, etomoxir, TTFA, antimycin A, rotenone, oligomycin A, MitoTempo and rapamycin) were purchased from Sigma. BCATi and nor-NOHA were purchased from Cayman Chemicals. Cell viability was assessed by Annexin-V and live–dead staining by FACS. Cells were counted at the microscope after trypan blue staining or, when in limited number, by FACS.

### Flow cytometry and cell sorting

Cells were stained with surface antibodies and Fixable Viability Dye eFluor 506 (eBioscience) in PBS supplemented with 2% fetal calf serum for 30 min on ice. For experiments involving intracellular staining of cytokines, cells were stimulated for 6 h with cytokines, and, during the last 3 h, Golgi Plug and Golgi Stop (BD) were added to the cultures. Cells were washed with PBS and fixed and permeabilized for 45 min at room temperature using a Cytofix/Cytoperm kit (BD). Intracellular staining was performed at room temperature for 30 min in the dark. Annexin-V staining was performed using Annexin-V-binding buffer (BD). Samples were acquired with an LSRFortessa (BD) and analyzed by FlowJ10.7.1 (TreeStar). For cell sorting, PBMCs were depleted of T cells, B cells, plasmacytoid dendritic cells, monocytes and erythrocytes by labeling with biotin-conjugated antibodies followed by anti-biotin microbeads and AutoMACS separation (Miltenyi) according to manufacturer’s instruction. Cells were sorted in bulk to a purity of ≥99% (FACSAria II; BD).

### Antibodies

Surface GLUT1 expression was monitored as a function of binding to its ligand, the envelope glycoprotein of the human T lymphotropic virus (HTLV). A recombinant HTLV envelope receptor-binding domain (H_RBD_) fused to an enhanced green fluorescent protein (eGFP)-coding sequence was used as previously described^56^. Surface ASCT2 was similarly evaluated; expression was monitored as a function of binding to its ligand, the RD114 envelope glycoprotein of the feline endogenous retrovirus, fused with a murine Fc tag and revealed with an Alexa Flour-647-conjugated anti-mouse IgG (Invitrogen).

Antibodies for ILC2 enrichment, including anti-hCD3 biotin (clone OKT3, 13-0037-82), anti-hCD4 biotin (RPA-T4, 13-0049-82), anti-hCD19 biotin (HIB19, 13-0199-82), anti-hCD14 biotin (61D3, 13-0149-82), anti-hCD123 biotin (6H6, 13-1239-82) and anti-hCD235a biotin (HIR2 GA-R2, 13-9987-82), were purchased from eBioscience. Antibodies used for ILC2 sorting, including anti-hCD3 FITC (UCHT1, 11-0038), anti-hCD4 FITC (OKT4, 11-0048), anti-hCD5 FITC (UCHT2, 11-0059), anti-hαβTCR FITC (IP26, 564451), anti-hγδTCR FITC (B1.1, 11-9986) and anti-hCD127 (IL-7Rα) PE-Cy7 (eBioRDR5, 25-1278-42) were purchased from eBioscience. Anti-hCD14 FITC (TUK4, 130-080-701), anti-hCD19 FITC (LT19, 130-104-650) and anti-hCD159a (NKG2A) PE (REA110, 130-113-566) were purchased from Miltenyi. Anti-hCD294 (CRTH2) Alexa Fluor 647 (BM16, 558042) and anti-hCD7 BV711 (M-T701, 564018) were purchased from BD. Anti-hCD45 AF700 (HI30, 560566), anti-hCD94 APC-Fire750 (DX22, 305518), anti-hCD117 BV605 (104D2, 313218), anti-hCD16 BV650 (3G8, 302042) and anti-hCD56 BV756 (5.1H11, 362550) were purchased from BioLegend. Antibodies for FACS analysis (extracellular and intracellular staining), including anti-hAnnexinV BV395 (564871), anti-hCD3 BUV737 (UCHT1, 612750), anti-hCD5 BUV737 (UCHT2, 612842), anti-hCD14 BUV737 (M5E2, 612763), anti-hCD19 BUV737 (SJ25C1, 612756), anti-hCD45 BV805 (HI30, 612891) and anti-hIL-13 BV421 (JES10-5A2, 624124), were purchased from BD. Anti-hST2 APC (hIL33Rcap, 17-9338-42) and anti-hAmphiregulin (AREG559, 17-5370-42) were purchased from eBioscience, and anti-hIL-5 (TRFK5, 504311) and anti-hHIF1α (546-16, 359704) were purchased from BioLegend.

### Mitochondrial mass, membrane potential by FACS and confocal microscopy and FA uptake

Mitochondrial mass, membrane potential and FA uptake of freshly sorted or cytokine-activated ILC2s were assessed by staining cells with 50 nM MitoTracker Green FM (Thermo Fisher), 25 nM TMRM (Sigma-Aldrich) and BODIPY FL-C_16_ (Thermo Fisher), respectively, for 30 min at 37 °C and 5% CO_2_. Cells were washed twice in cold 1× PBS, stained with surface antibodies and analyzed by FACS. For confocal microscopy, cells were stained at 37 °C for 30 min with 300 ng ml^–1^ of Hoechst H33342 (Life Technologies) to stain nuclei, 100 nM MitoTracker Green FM to stain mitochondria and 25 nM TMRM to asses mitochondrial membrane potential (non-quenching mode, TMRM maintained in the cell medium). Cells were plated in a 384-well plate (40,000 cells per well), and image acquisitions of multiple fields per well were performed on an automated confocal microscope (OPERA QEHS, Perkin Elmer) using ×60 objectives, excitation lasers at 405, 488 and 561 nm and emission filters at 450, 540 and 600 nm, respectively. Confocal images were transferred to the Columbus Image Data Storage and Analysis System (Perkin Elmer) for high content analyses as previously reported^57^ and used the standard deviation/mean approach^58^.

### Metabolite extraction, mass spectrometry and data analysis

Methods for metabolite extraction, data acquisition and data analysis were developed and performed by General Metabolics. FACS-sorted ILC2s (100,000 cells) were pelleted and washed in prewarmed (37 °C) ammonium carbonate (75 mM) washing buffer. Preheated (70 °C) 70% ethanol (99.9% purity) extraction solvent was added, and samples were incubated for 3 min. After centrifugation (8,000*g* for 10 min at 0 °C), the supernatants were collected and stored at −80 °C until analysis. The analysis was performed on a platform consisting of an Agilent Series 1100 LC pump coupled to a Gerstel MPS2 autosampler and an Agilent 6520 Series Quadrupole time-of-flight mass spectrometer equipped with an electrospray source operated in negative and positive mode as previously reported^59^. All steps of data processing and analysis were performed with Matlab R2010b (MathWorks) using functions embedded in the bioinformatics, statistics, database and parallel computing toolboxes. For each run, a matrix list was produced with the intensity of each mass peak in each analyzed sample. An accurate *m*/*z* was recalculated with a weighted average of the values, and a list of putative metabolites was compiled from the KEGG database. Raw intensities for each metabolite are included in Supplementary Data 1. For each ion, the best metabolite match was chosen among all candidates based on the deviation from the theoretical *m*/*z* and a heuristic probability associated with the theoretical ion fragment, which was set lower for, for example, rare adducts, fragments or molecules containing several heteroatoms. Significant changes in metabolite levels compared to the entire dataset were determined by calculating *z* scores for each sample and ion individually, as previously reported^56^. Results were run in iPath3 (https://pathways.embl.de), an online tool for data mapping. A Principal-component analysis was applied to the samples coming from two different screenings using the stats R package. The screenings were simultaneously analyzed considering only metabolites present in both screenings (total = 122 metabolites). The first principal component indicated that most of the variance of the data corresponded to the screening effect, as expected from the batch effect that each screening represented. The second most variable effect corresponded to the donors. Pathway analysis was done with Qlucore Omic Explorer v3.

### RNA isolation, library construction, sequencing and analysis

ILC2s (10^3^ cells) were sorted by FACS directly into 50 ml of lysis/binding buffer (Life Technologies). mRNA was captured with 15 ml of Dynabeads oligo(dT) (Life Technologies), washed according to manufacturer’s instructions and eluted at 85 °C with 6 ml of 10 mM Tris-HCl (pH 7.5). We used a derivation of MARS-seq as described in ref. ^60^, developed for single-cell RNA-seq, to produce expression libraries of two replicates per population. Libraries were sequenced at an average depth of 5 million reads per library on an Ilumina NextSeq and aligned to the human reference genome (hg19). Reads were mapped using hisat (version 0.1.6); duplicate reads were filtered if they had identical unique molecular identifiers. Expression levels were calculated and normalized to the total number of reads using HOMER software. RNA-seq datasets have been deposited in the Gene Expression Omnibus (GEO) public repository (accession number GSE183669).

### Transcriptional profiling by BioMark

ILC2s were sorted as small bulks (25 cells) directly in 96-well qPCR plates with RT mix 1 (5× VILO Reaction Mix, 200 U µl^–1^ SUPERase-In, 10% NP-40 and nuclease free water). Reverse transcription was performed according to manufacturer’s protocols (Fluidigm). The dynamic Array IFC chip was prepared according to manufacturer’s protocols and analyzed in the Biomark System (Fluidigm). Cycle threshold (*C*_t_) values were collected from the machine and processed using Prism 8.

### Cellular metabolism by Seahorse extracellular flux analyzer

OCRs and ECARs were measured for freshly sorted, IL-2-, IL-7- and/or IL-33-stimulated ILC2s (100,000 cells). XF medium (non-buffered RPMI 1640 containing 10 mM glucose, 2 mM l-glutamine and 1 mM sodium pyruvate) was used under basal conditions. Addition of 1 μM oligomycin, 1.5 μM FCCP and 100 nM rotenone + 1 μM antimycin A was performed using portal injection in a 96-well XF or XFe Extracellular Flux Analyzer (Seahorse Bioscience).

### Statistical analysis

Flow cytometry data were analyzed using FlowJo v.10 (TreeStar). Statistical analyses were done using a two-tailed *t*-test or one-way ANOVA test with Dunnett correction when comparing multiple groups to specific conditions or a Tukey correction for multiple comparisons when comparing more than two conditions of interest (GraphPad Prism v.8 and v.9). The statistical tests, replicate experiments and *P* values are all cited in the figures and/or figure captions. Statistical tests were justified as appropriate for every figure, and the data meet the assumptions of the tests. The ranges of *x* and *y* axes for scatter plots were determined to include all of the data points. The sample size for each experiment and the replicate number of experiments are included in the figure legends as well as the specific test used for the analysis.

### Reporting Summary

Further information on research design is available in the Nature Research Reporting Summary linked to this article.

## Online content

Any methods, additional references, Nature Research reporting summaries, source data, extended data, supplementary information, acknowledgements, peer review information; details of author contributions and competing interests; and statements of data and code availability are available at 10.1038/s41590-021-01043-8.

## Supplementary information

Reporting Summary

Peer Review Information

Supplementary Table 1 Descriptions of individuals with MD.

Supplementary Data 1 Mass spectrometry datasets.

Supplementary Data 2 RNA-seq datasets.

## Source data

Source Data Fig. 1 Source data for the presented plots.

Source Data Fig. 2 Source data for the presented plots.

Source Data Fig. 3 Source data for the presented plots.

Source Data Fig. 4 Source data for the presented plots.

Source Data Extended Data Fig. 1 Source data for the presented plots.

Source Data Extended Data Fig. 1 Unprocessed confocal microscopy images.

Source Data Extended Data Fig. 2 Source data for the presented plots.

Source Data Extended Data Fig. 3 Source data for the presented plots.

Source Data Extended Data Fig. 4 Source data for the presented plots.

Source Data Extended Data Fig. 6 Source data for the presented plots.

Source Data Extended Data Fig. 7 Source data for the presented plots.

Source Data Extended Data Fig. 8 Source data for the presented plots.

Source Data Extended Data Fig. 9 Source data for the presented plots.

## Data Availability

All the data generated and/or analyzed during this study are included in this manuscript as Supplementary Information. RNA-seq datasets have been deposited in the GEO public repository (accession number GSE183669). Source data are provided with this paper.
