# Supplementary material for: Dichotomous metabolic networks govern human ILC2 proliferation and function
Source: Nat Immunol. 2021 Oct 22;22(11):1367–74. doi: 10.1038/s41590-021-01043-8 (PMC8553616; doi:10.1038/s41590-021-01043-8)
Supplement: Supplementary file 1 — Reporting Summary [file 41590_2021_1043_MOESM1_ESM.pdf]

nature research

Corresponding author(s): James Di Santo

Last updated by author(s): Aug 31, 2021

Reporting Summary

Nature Research wishes to improve the reproducibility of the work that we publish. This form provides structure for consistency and transparency in reporting. For further information on Nature Research policies, see our Editorial Policies and the Editorial Policy Checklist.

Statistics

For all statistical analyses, confirm that the following items are present in the figure legend, table legend, main text, or Methods section.

n/a	Confirmed	
☐	☒	The exact sample size (*n*) for each experimental group/condition, given as a discrete number and unit of measurement
☐	☒	A statement on whether measurements were taken from distinct samples or whether the same sample was measured repeatedly
☐	☒	The statistical test(s) used AND whether they are one- or two-sided<br>*Only common tests should be described solely by name; describe more complex techniques in the Methods section.*
☐	☒	A description of all covariates tested
☐	☒	A description of any assumptions or corrections, such as tests of normality and adjustment for multiple comparisons
☐	☒	A full description of the statistical parameters including central tendency (e.g. means) or other basic estimates (e.g. regression coefficient) AND variation (e.g. standard deviation) or associated estimates of uncertainty (e.g. confidence intervals)
☐	☒	For null hypothesis testing, the test statistic (e.g. *F*, *t*, *r*) with confidence intervals, effect sizes, degrees of freedom and *P* value noted<br>*Give P values as exact values whenever suitable.*
☒	☐	For Bayesian analysis, information on the choice of priors and Markov chain Monte Carlo settings
☒	☐	For hierarchical and complex designs, identification of the appropriate level for tests and full reporting of outcomes
☐	☒	Estimates of effect sizes (e.g. Cohen's *d*, Pearson's *r*), indicating how they were calculated

Our web collection on statistics for biologists contains articles on many of the points above.

Software and code

Policy information about availability of computer code

Data collection	no software was used
Data analysis	Qlucore Omic Explorer v3, Prism8, FlowJo v10, Matlab R2010b, Hisat (version 0.1.6), HOMER

For manuscripts utilizing custom algorithms or software that are central to the research but not yet described in published literature, software must be made available to editors and reviewers. We strongly encourage code deposition in a community repository (e.g. GitHub). See the Nature Research guidelines for submitting code & software for further information.

Data

Policy information about availability of data

All manuscripts must include a data availability statement. This statement should provide the following information, where applicable:

- Accession codes, unique identifiers, or web links for publicly available datasets
- A list of figures that have associated raw data
- A description of any restrictions on data availability

All data generated or analysed during this study are included in this published article (as supplementary information files). RNA-seq datasets have been deposited in the GEO public repository (accession number GSE183669).

Field-specific reporting

Please select the one below that is the best fit for your research. If you are not sure, read the appropriate sections before making your selection.

- [x] Life sciences
- [ ] Behavioural & social sciences
- [ ] Ecological, evolutionary & environmental sciences

For a reference copy of the document with all sections, see nature.com/documents/nr-reporting-summary-flat.pdf

Life sciences study design

All studies must disclose on these points even when the disclosure is negative.

Sample size	Sample size was based on donor availability.
Data exclusions	No data were excluded from this study.
Replication	Each experiment published in this study has been repeated at least 2 times (indicated in the figure legends). Crucial experiments were performed and analyzed interdependently by the first and second authors.
Randomization	Allocation was random for each experiment. Donors were assigned numbers and shuffled at the beginning of the experiment.
Blinding	confocal microscopy was done in a single blinded fashion. Other experiments ere not done in a blind fashion as the investigators need to design, conduct and analyze the data, thus they need to know the identification of samples.

Reporting for specific materials, systems and methods

We require information from authors about some types of materials, experimental systems and methods used in many studies. Here, indicate whether each material, system or method listed is relevant to your study. If you are not sure if a list item applies to your research, read the appropriate section before selecting a response.

Materials & experimental systems

n/a	Involved in the study	
[ ]	[x]	Antibodies
[x]	[ ]	Eukaryotic cell lines
[x]	[ ]	Palaeontology and archaeology
[x]	[ ]	Animals and other organisms
[ ]	[x]	Human research participants
[x]	[ ]	Clinical data
[x]	[ ]	Dual use research of concern

Methods

n/a	Involved in the study	
[x]	[ ]	ChIP-seq
[ ]	[x]	Flow cytometry
[x]	[ ]	MRI-based neuroimaging

Antibodies

Antibodies used	Surface Glut1 expression was monitored as a function of binding to its ligand, the envelope glycoprotein of the human T lymphotropic virus (HTLV). A recombinant HTLV envelope receptor-binding domain (HRBD) fused to an EGFP coding sequence was used as previously described (Manel et al. 2003). Surface ASCT2 was similarly evaluated; expression was monitored as a function of binding to its ligand, the RD114 envelope glycoprotein of the feline endogenous retrovirus (Laval et al; 2013), fused with a murine Fc tag and revealed with an Alexa-Flour-647-conjugated anti-mouse IgG (Invitrogen).<br>Antibodies for ILC2 enrichment anti-hCD3 biotin (Clone OKT3, Cat#13-0037-82, 1/100), anti-hCD4 biotin (RPA-T4, 13-0049-82, 1/100), anti-hCD19 biotin (HIB19, 13-0199-82, 1/100), anti-hCD14 biotin (61D3, 13-0149-82, 1/100), anti-hCD123 biotin (6H6, 13-1239-82, 1/100) and anti-hCD235a biotin (HIR2 GA-R2, 13-9987-82, 1/100) were purchased from eBioscience. Antibodies used for ILC2 sorting anti-hCD3 FITC (UCHT1, 11-0038, 1/100), anti-hCD4 FITC (OKT4, 11-0048, 1/100), anti-hCD5 FITC (UCHT2, 11-0059, 1/100), Anti-hαβTCR FITC (IP26, 564451, 1/100), Anti-hγδTCR FITC (B1.1, 11-9986, 1/100), anti-hCD127 (IL-7Ra) PE-Cy7 (eBioRDR5, 25-1278-42, 1/50) were purchased from eBioscience. Anti-hCD14 FITC (TUK4, 130-080-701, 0,5/100), anti-hCD19 FITC (LT19, 130-104-650, 0,5/100), Anti-hCD159a (NKG2A) PE (REA110, 130-113-566, 1/500) were purchased from Miltenyi. Anti-hCD294 (CRTH2) Alexa Fluor 647 (BM16, 558042, 1/25), anti-hCD7 BV711 (M-T701, 564018, 1/100) were purchased from BD. Anti-hCD45 AF700 (HI30, 560566, 1/100), anti-hCD94 APC-Fire750 (DX22, 305518, 1/25), anti-hCD117 BV605 (104D2, 313218, 1/50), anti-hCD16 BV650 (3G8, 302042, 1/100), anti-hCD56 BV756 (5.1H11, 362550, 1/100) were purchased from Biolegend. Antibodies for FACS analysis (extracellular and intracellular staining) anti-hAnnexinV BV395 (564871, 1/100), anti-hCD3 BUV737 (UCHT1, 612750, 1/100), anti-hCD5 BUV737 (UCHT2, 612842, 1/100); anti-hCD14 BUV737 (M5E2, 612763, 1/100), anti-hCD19 BUV737 (SJ25C1, 612756, 1/100), anti-hCD45 BV805 (HI30, 612891, 1/200), anti-hIL-13 BV421 (JES10-5A2, 624124, 1/100) were purchased from BD, anti-hST2 APC (hIL33Rcap, 17-9338-42, 1/25) and anti-Amphiregulin (AREG559, 17-5370-42, 1/50) were purchased from ebioscience, anti-hIL-5 (TRFK5, 504311, 1/100) and anti-hHIF1a (546-16,359704, 1/100) were purchased from biolegend.<br>Antibodies were validated by the supplier.

Validation	Antibodies were validated by the supplier (commercially available) or by collaborators: GLUT1 (Manel et al. 2003) and ASCT2 (Laval et al; 2013).

Human research participants

Policy information about studies involving human research participants

Population characteristics	Healthy donors included in the study were randomly selected (age, sex). The EFS (Establissement Francais du Sang) did not share information about clinical history or past diagnosis. Mitochondrial disease patients were selected based on the diagnosis (PEO, ADOA, MELAS, PEO+AO), but randomly selected for age and sex. All the patients had genetic mutations as leading cause for mitochondrial disease (described in the table). No clinical history file has been shared by the clinician.
Recruitment	Healthy donors were randomly recruited by the Establissement Francais du Sang (EFS, Paris). PBMC from mitochondrial disease patients were obtained from Unit of Neurology and Neuromuscular Disorders at "University of Messina".
Ethics oversight	All studies required institutional ethics committee approval.

Note that full information on the approval of the study protocol must also be provided in the manuscript.

Flow Cytometry

Plots

Confirm that:

☒ The axis labels state the marker and fluorochrome used (e.g. CD4-FITC).

☒ The axis scales are clearly visible. Include numbers along axes only for bottom left plot of group (a 'group' is an analysis of identical markers).

☒ All plots are contour plots with outliers or pseudocolor plots.

☒ A numerical value for number of cells or percentage (with statistics) is provided.

Methodology

Sample preparation	Isolation of the human peripheral blood mononuclear cells (PBMC) and single cell suspension was achieved by Ficoll-Paque (GE Healthcare) density gradient centrifugation.<br>Cells were stained with surface antibodies and Flexible Viability Dye eFluor 506 (eBioscience) in PBS 2%FCS for 30 minutes on ice. For experiments involving intracellular staining of cytokines, cells were stimulated for 6 hours with cytokines and during the last 3 hours Golgi Plug and Golgi Stop (BD) were added to the cultures. Cells were washed with PBS and fixed/ permeabilized for 45 minutes at RT by Cytofix/Cytoperm kit (BD). Intracellular staining was performed at RT for 30 minutes in the dark. AnnexinV staining was performed using the Annexin V binding buffer (BD). Samples were acquired at the LSRFortessa (BD) and analyzed by FlowJ10.7.1 (Tree Star). For cell sorting PBMC were depleted of T cell, B cells, pDC, monocytes and erythrocytes by labeling with biotin conjugated antibodies, followed by anti-biotin microbeads and AutoMACS separation (Miltenyi) according to manufacturer's instruction. Cells were sorted in bulks to a purity of ≥99% (FACSAria II; BD).
Instrument	LSRFortessa (BD), (FACSAria II; BD)
Software	FlowJo, Diva
Cell population abundance	Cells were sorted in bulks to a purity of ≥99% (screened by FACS) or as single cell index sorting (FACSAria II score)
Gating strategy	ILCs Gating strategy: Live CD45+ CD7+ Lin- CD94- NKG2A- CD56- CD16- CD127+ CRTh2+ (ILC2) and Live CD45+ CD7+ Lin- CD94+ CD16+ (NKDim).

☒ Tick this box to confirm that a figure exemplifying the gating strategy is provided in the Supplementary Information.
