# Supplementary material for: Dichotomous metabolic networks govern human ILC2 proliferation and function
Source: Nat Immunol. 2021 Oct 22;22(11):1367–74. doi: 10.1038/s41590-021-01043-8 (PMC8553616; doi:10.1038/s41590-021-01043-8)

NI-LE31657C_SourceData_ED_Fig1_Image1_Hoechst

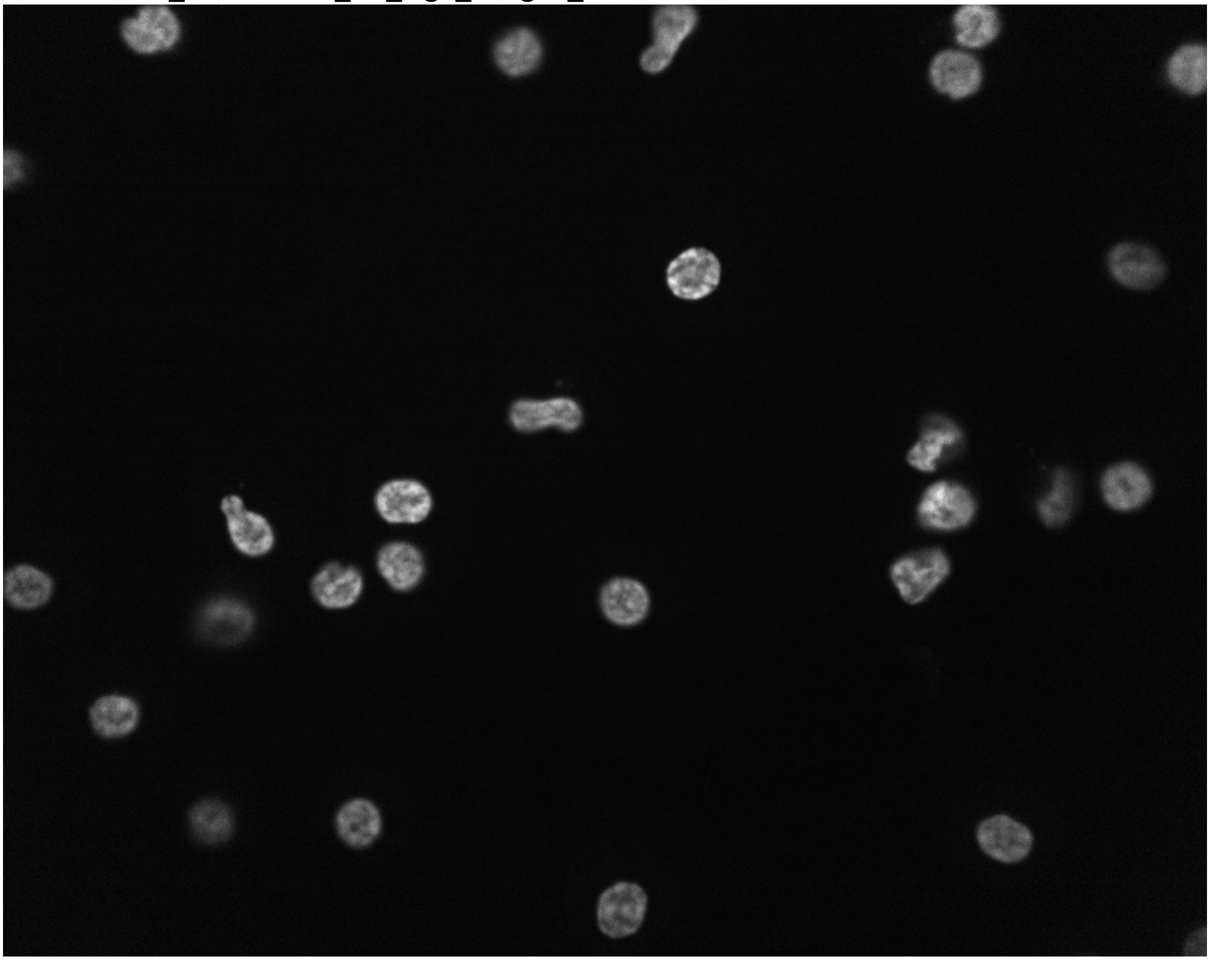

NI-LE31657C_SourceData_ED_Fig1_Image2_MItotracker

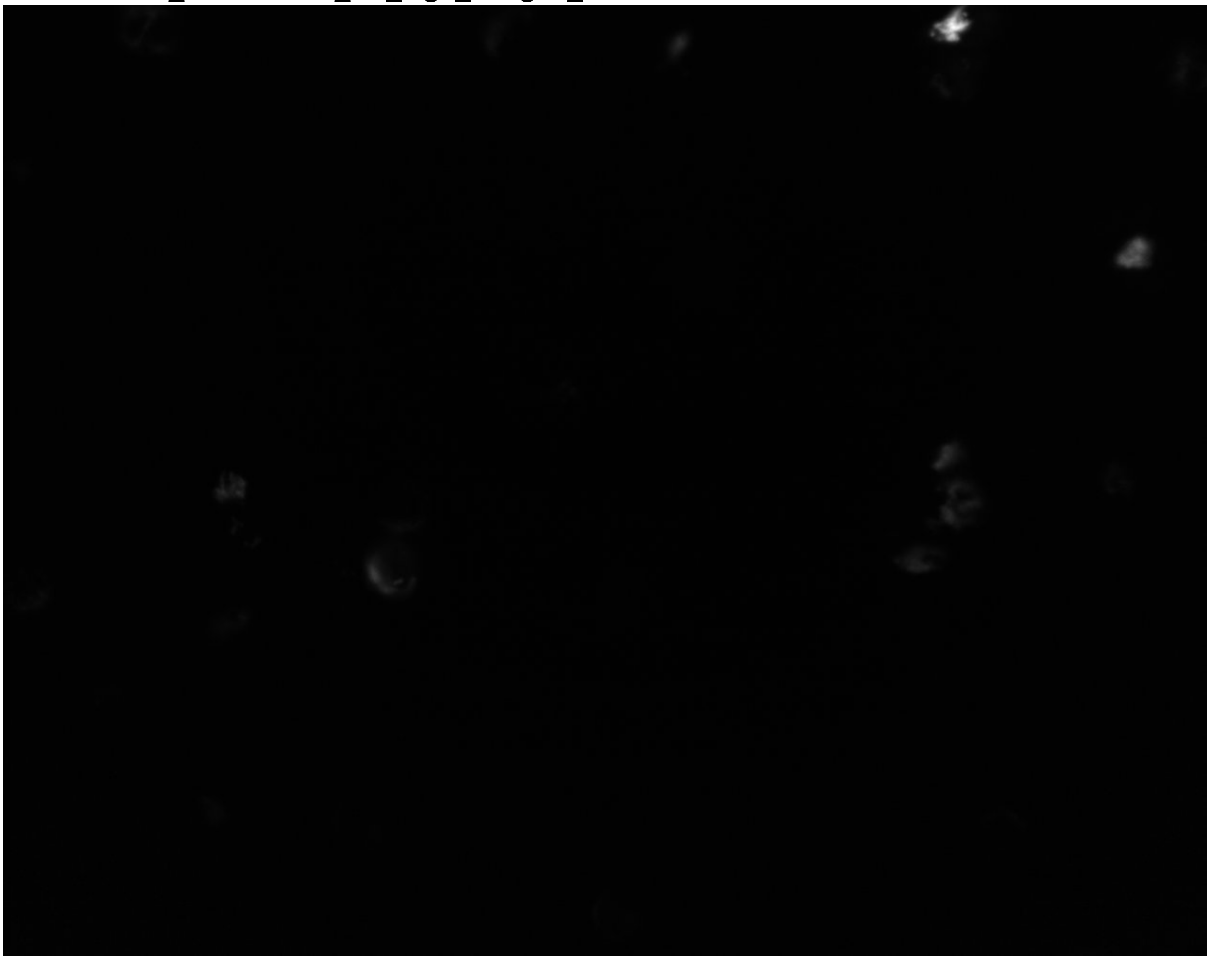

NI-LE31657C_SourceData_ED_Fig1_Image3_TMRM

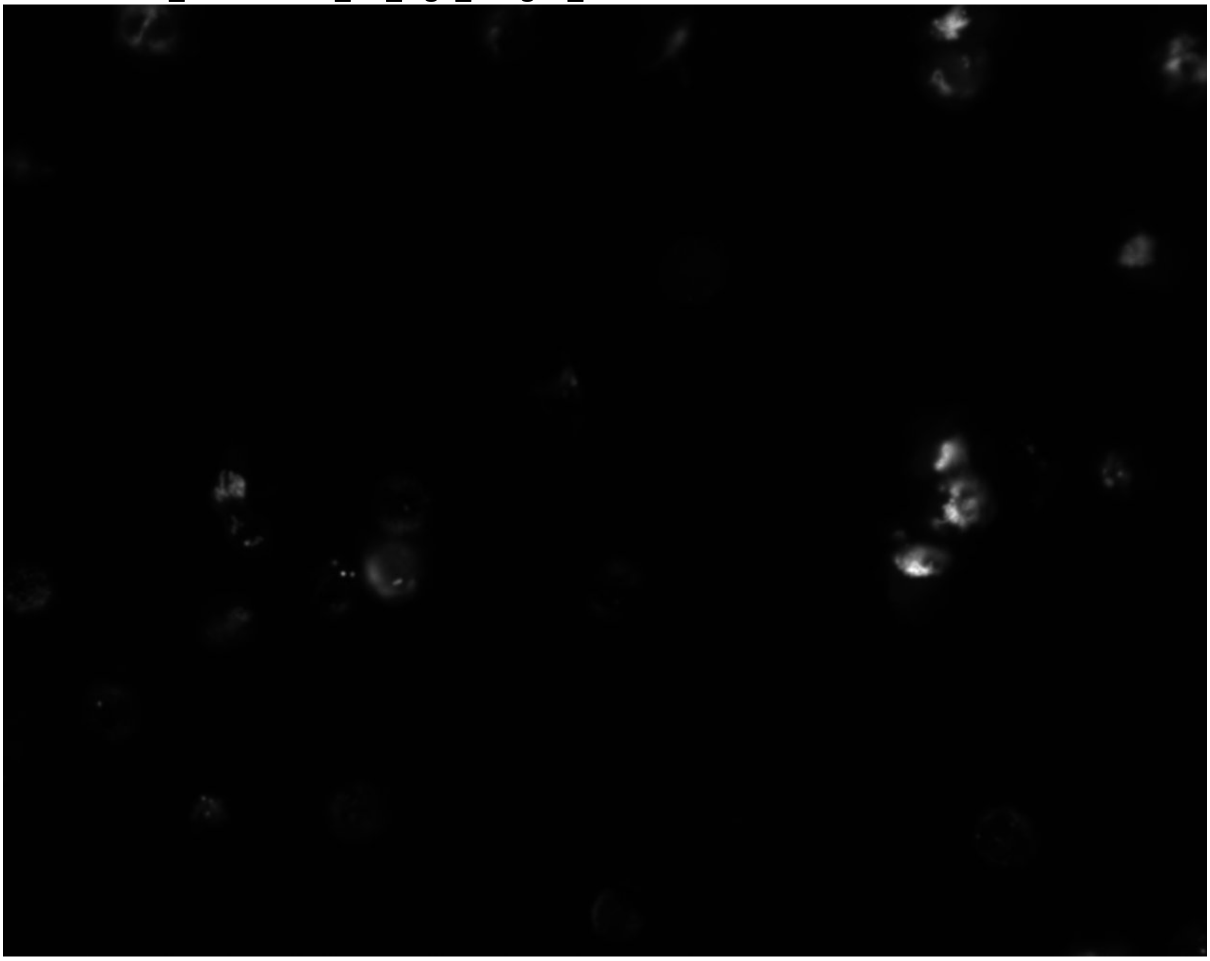

NI-LE31657C_SourceData_ED_Fig1_Image4_Hoechst

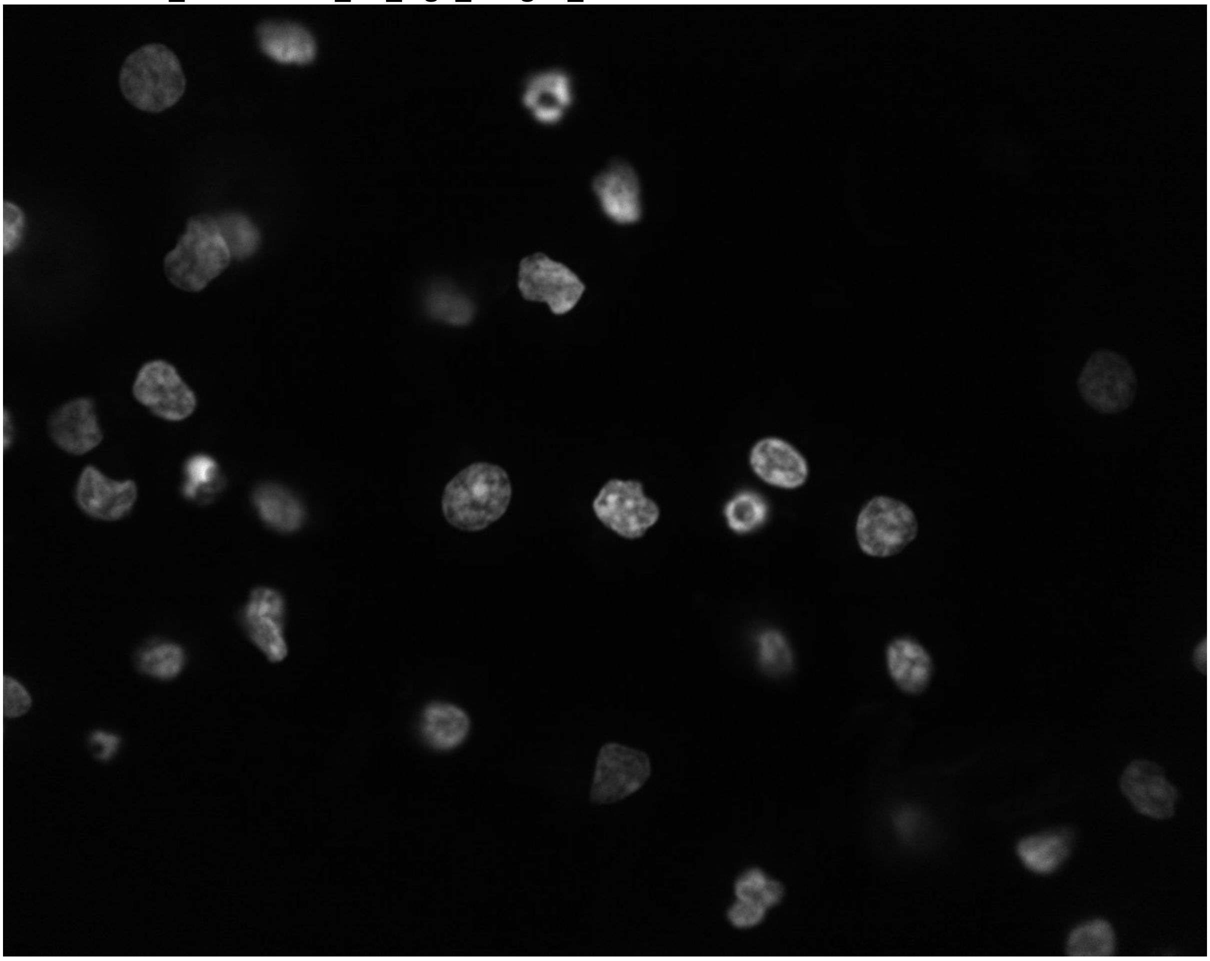

NI-LE31657C_SourceData_ED_Fig1_Image5_TMRM

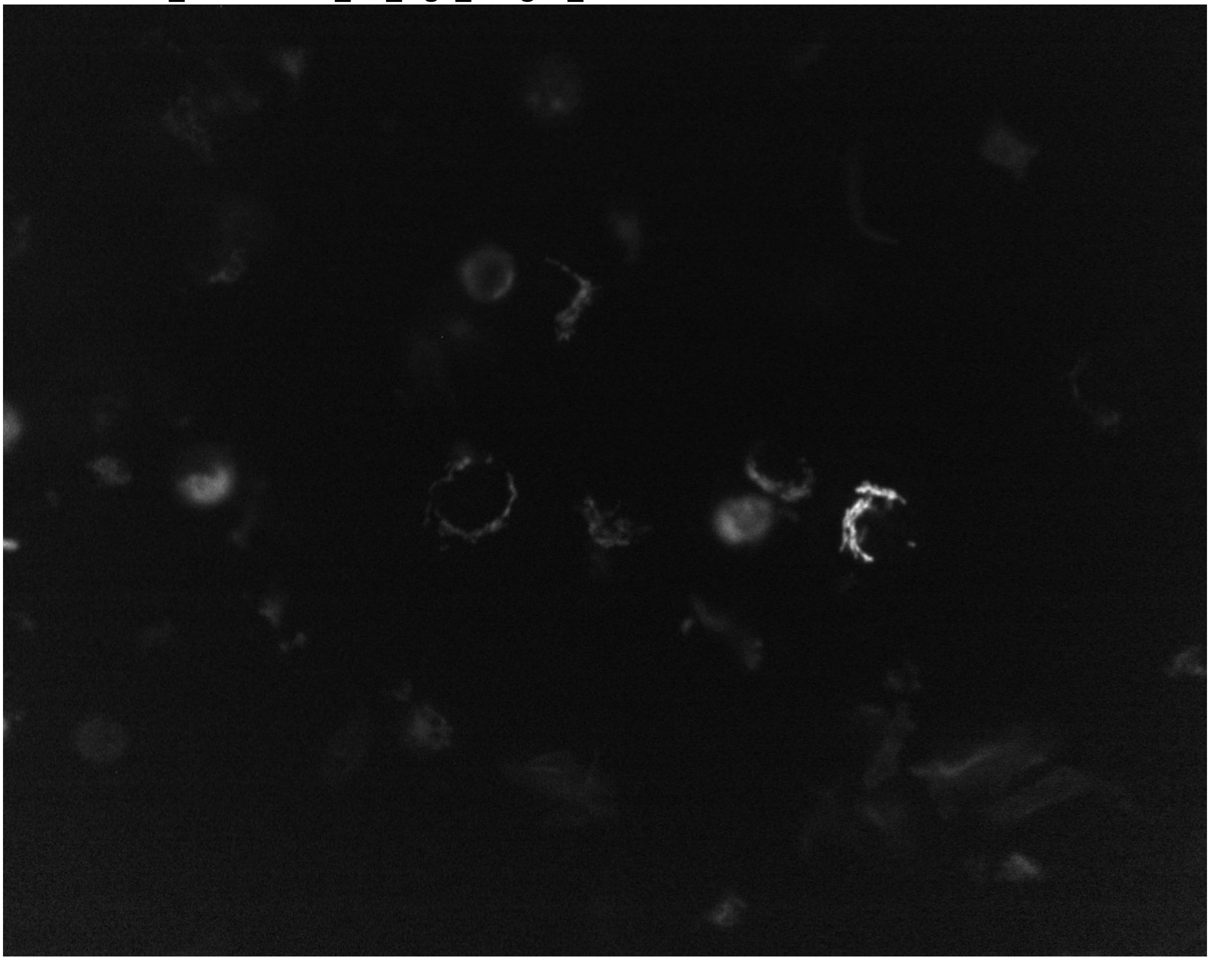

NI-LE31657C_SourceData_ED_Fig1_Image6_Mitotracker

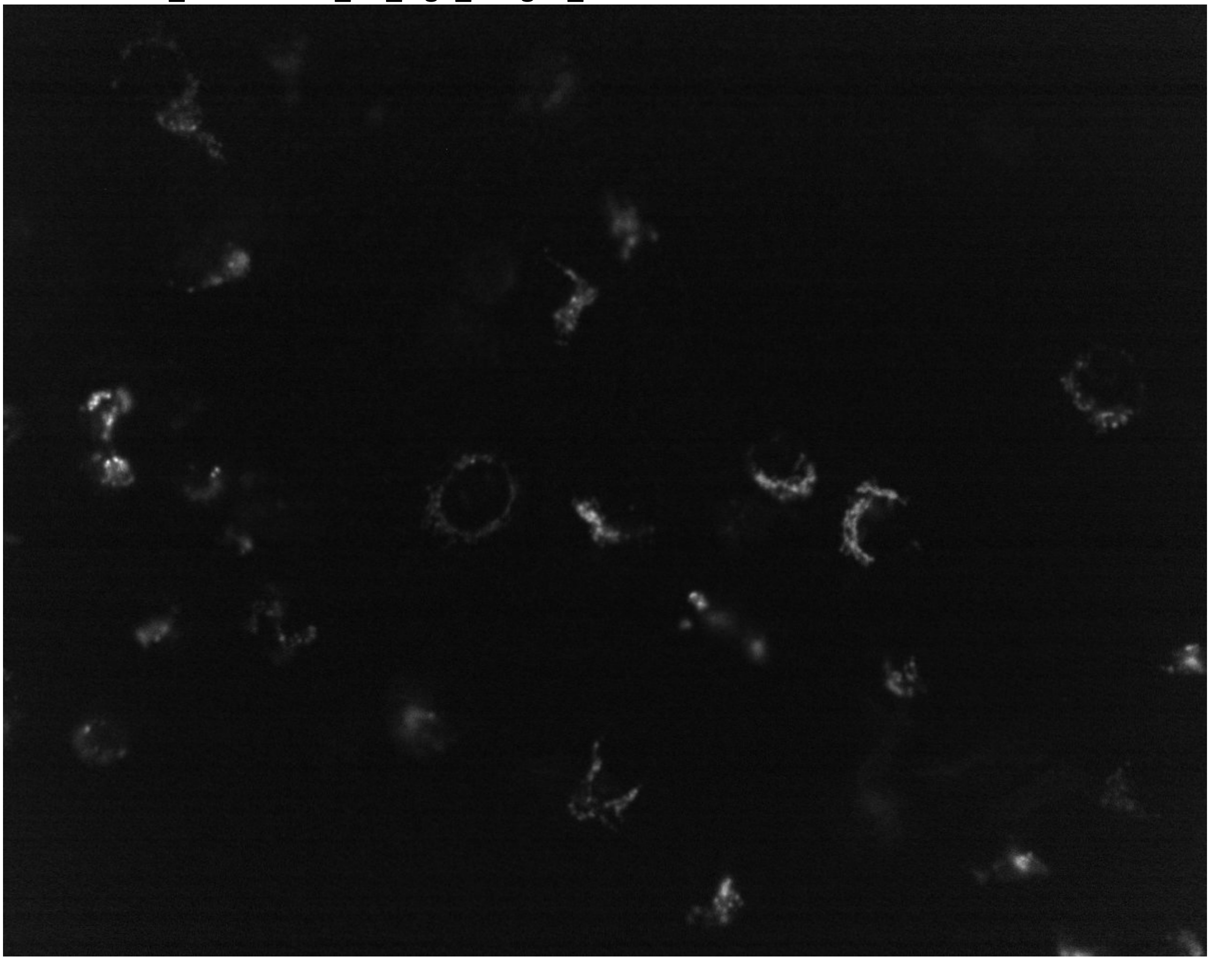

Supplement: Supplementary file 11 — Unprocessed confocal microscopy images. [file 41590_2021_1043_MOESM11_ESM.pdf]
