# Supplementary material for: Dichotomous metabolic networks govern human ILC2 proliferation and function
Source: Nat Immunol. 2021 Oct 22;22(11):1367–74. doi: 10.1038/s41590-021-01043-8 (PMC8553616; doi:10.1038/s41590-021-01043-8)
Supplement: Supplementary file 2 — Peer Review Information [file 41590_2021_1043_MOESM2_ESM.pdf]

natureresearch

Peer Review Information

Journal: Nature Immunology
Manuscript Title: Dichotomous metabolic networks govern human ILC2 proliferation and function
Corresponding author name(s): James P. Di Santo

Editorial Notes:

**Redactions – unpublished data**	Parts of this Peer Review File have been redacted as indicated to maintain the confidentiality of unpublished data.

Reviewer Comments & Decisions:

Decision Letter, initial version:

Subject: Decision on Nature Immunology submission NI-LE31657
Message: 24th Mar 2021

Dear Jim,

We have now finished reviewing your manuscript entitled "Dichotomous metabolic networks govern human ILC2 proliferation and function", reference number NI-LE31657.

Although the editors thought that the manuscript was interesting enough to send out for in-depth review, the reviewers were not in favor of publishing the paper in Nature Immunology, as all three voiced that they found the study to be too preliminary. They note that better comparisons are needed for the circulating ILC2 cells, eg to ILCp and/or tissue-resident ILC2. They have also voiced questions about the role of hypoxia, examination of other cytokines, heterogeneity in the circulating ILC2 population, etc. Additionally, more information is also needed about their human donors - especially those with mitochondrial diseases.

Based on the referees' advice, we decided to reject the current manuscript, but we would be willing to consider a resubmission if you are able to extend the study along the lines suggested by the referees. Please let us know if you wish to pursue this route and if so how long you anticipate the experimental revisions would require to complete.

I hope that you continue to consider Nature Immunology for your results most significant for the immunology community and wish you well in your future investigations.

Sincerely,

Laurie

Laurie A. Dempsey, Ph.D.
Senior Editor
Nature Immunology
l.dempsey@us.nature.com
ORCID: 0000-0002-3304-796X

Reviewers' comments:

Reviewer #1 (Remarks to the Author):

In this manuscript the Di Santo group investigated the metabolic requirements of human group 2 innate lymphoid cell (ILC2). Data presented by the authors here suggest that circulating "naïve" ILC2 have a higher level of oxidative phosphorylation (OXPHOS) than other ILC such as NK cell. Interestingly, ILC2 are severely reduced in patients with mitochondrial disease and impaired OXPHOS. Metabolomic and nutrient receptors analysis further demonstrated that ILC2 take up amino acids to sustain OXPHOS at steady-state. Upon activation with IL-33, ILC2 become highly proliferative relying on glycolysis and mTOR to produce IL-13, while continuing to fuel OXPHOS with amino acids to maintain cellular fitness and proliferation. Based on these observations the authors suggest that proliferation and function are metabolically uncoupled in human ILC2, offering new strategies to target ILC2 in disease settings.
The manuscript by Di Santo and colleagues is well put together and address key questions in the field of ILC2 biology. It is a refreshing read performed with human ILC2 and I congratulate the authors for this nice piece of work. However, while the presented set of data is very intriguing, essential controls are lacking and a big portion of claims are poorly justified by the presented data in its current form. Below major and some more specific comments are listed to support the authors to bring that manuscript to fruition and publication.

Major comments
1. The authors claim that their presented data allows the conclusion that proliferation and function are metabolically uncoupled in human ILC2. This reviewer respectfully disagrees as single cytokine experimental controls are lacking throughout the manuscript. Throughout the manuscript the authors rely on the sole comparison of IL-2+IL-7+IL-33 and IL-2+IL-7. Single cytokine stimulations need to be performed to distinguish the effects of IL-2 from IL-7, but most importantly to compare IL-33 only vs IL-33+IL-2, IL-33+IL-7 or IL-33+IL-2+IL-7.
2. When major experiments involve addition of various metabolic inhibitors the authors the show side-by-side, intracellular cytokine production, # of cells, proliferation as well as cell death. Do alterations of metabolic pathways lead to either, changes in cytokine production, altered proliferation, and/or cell death? These questions especially need to be addressed when altering oxygen availability (e.g. Figure 3E).
3. Throughout the manuscript the author mostly analyzed IL-13 levels as a readout for

cytolone production. Have the authors looked at other ILC2-producing cytokines such as IL-5, IL-6, and GM-CSF? In addition, has the production of anti-inflammatory and tissue restorative cytokines including IL-10 and Areg been explored? It will be important to test this to highlight of whether inflammatory as well as anti-inflammatory mediators have similar or distinct metabolic requirements.
4. Since this is a human study using patients as well as healthy donors, a table showing the clinicopathological data is warranted. Have the authors stratified patients by age, sex, and ethnic background (although it can be assumed that the patients and healthy donors are predominantly Caucasians) to determine if there are any differences seen in ILC2 levels?
5. The authors state that ILC2s are severely reduced in "Mitochondrial Disease". Mitochondrial Disease is an umbrella term encompassing various diseases such as Mitochondrial Myopathy and MERRF Syndrome, ….. Can the authors show a breakdown of patients with the various diseases and to possibly stratify patients according to disease type and specific gene alteration to determine any differences.
6. At the end of their manuscript the authors put forward the sweeping claim that "FA do not sustain bioenergetic needs but might accumulate upon cell division". This reviewer does not think that the authors present supportive evidence for this sweeping claim. (i) Although ETO treatment presented in data presented in Ext Fig. 4e does not show a statistically significant difference it could depend on does and kinetic of ETO treatment; (ii) FA oxidation could rely on peroxisomes rather than mitochondria; (iii) FA uptake could be decoupled from FA accumulation as liponeogenesis pathways could play a role here as well. (iv) Experiments restricting Glucose and or FA concentration in media would address this angle here.
As such I recommend the authors to discuss these aspects more carefully.
7. Do the authors think that blood ILC2 behave metabolically similar to tissue ILC2? Can this aspect be at least touched on upon experimentally or discussed?

Specific comments
1. Either table 1 was lacking or was mislabelled as a Figure, but this reviewer did not have access to data referred to as Table 1.
2. Figure 1B: Frequencies need to be supplemented by absolute cell counts to allow conclusions made by the authors (e.g. number of ILC2 in relation to total CD45-positve cells).
3. Fig 1 showed flow cytometry plots showing Mitotracker and TMRM and looking at ILC2s vs NKDim. Unstained Control peaks should be included.
4. ILC2 gating strategy (Supp Fig 1A) showed CD94-NKG2A-, although the supposed double negative population looks are and entering the 103 scale. It might help the authors to extend the scales and provide a FMO to ensure proper gating.
5. Fig 1 showed MFI. Was it mean or median? While knowing that the peaks are normally distributed is an important factor for MFI, perhaps it might help the authors to use Geometric MFI (gMFI) for their analysis?
6. Fig 1E looks like the NKDim and ILC2s are acquired on separate events, or could it be a compensation issue since the double negative populatons look different between the 2 cell types.
7. Fig 2b the authors performed RNASeq on 3 healthy donors. It might help the authors to do RNASeq on the patient blood and provide a heatmap looking at different gene expression? Do these transporters change after combined activation with IL2/IL-7+IL-33? It would be important to shed light on this? Can RNA-Seq data be complemented by flow cytometric data of some of the highest expression transporters?
8. Fig 2 the authors used nor-NOHA, BCATi, BPTES, and 2DG at 1μM. How did the authors

determine optimal concentrations? Can they provide a titration curve, or a dose-response curve to explain why 1μM was used?
9. Fig 3A the authors state that cell counts were determined by FACS. Was it by counting beads, or just by regular gatings? Will trypan blue and a haemocytometer validate those results? Please give details in the methods sections.
10. Fig 3 showed expansion of ILC2s for 7 days with IL-2 + IL-7 and then stimulated with or without IL-33 for 72 hours. Did the authors do any cytokine or serum starvation prior to IL-33 stimulation, to ensure the cells were back to baseline levels?
11. Fig 3E x-axis label to include Oxygen level
12. Fig 3E, have the authors looked at other ILC2 activation and co-stimulatory markers such as ICOS, KLRG1, Thy1, and CD25 or CD127?
13. Figs 4D and 4E no stats for D6?

Reviewer #2 (Remarks to the Author):

The manuscript by Surace et al. presents a metabolic characterization of human circulating ILCs, using freshly sorted ILC2s and CD56+CD16+NKDim cells, as well as ILC2s exposed to various cytokines in vitro. The authors show that circulating ILC2s have relatively enhanced mitochondrial function as compared to NK cells. In peripheral blood of patients with mitochondrial disorders, the frequency of ILC2s are reduced, and exhibit lowered mitochondrial mass/polarization, decreased GATA-3 staining and increased Annexin-V staining, suggesting that ILC2 function and survival are impaired in the setting of MD. In support of this, inhibition of ETC complex I and III in cultured ILC2s impaired survival, whereas NKDim cells were unaffected. Metabolomic analysis indicated an enrichment of arginine and branched amino acids (BCAA) in ILC2s along with the membrane transporters LAT1 and LAT4, while inhibition of arginase-1 and BCAT pathways reduced mitochondria and OXPHOS and altered corresponding transcriptional pathways in ILC2s. Culturing sorted ILC2s with IL-2/7/33 increased OCR and DSRC compared to IL-2/7 alone, and inhibition of ETC complex I and III impaired ILC2 survival. The authors suggest that ILC2s maintain both OXPHOS and enhanced glycolytic pathways during activation to maintain both cell fitness and IL-13 production, respectively.
This manuscript is clear and concisely written, adding an important analysis of human ILC2 metabolism to the field. The observations are somewhat limited in this brief report; however, particularly with respect to issues of development/tissue residency that are raised in the text. Specifically:
1. The authors draw attention to the unique and 'non-naïve' metabolic profile of circulating ILC2s, but compare only to NKDim cells, which they previously compared with NKbright cells in a recent publication describing similar relative differences (Blood Adv (2021) 5 (1): 26–38). It is difficult to get a sense of what makes the ILC2 metabolic profile unique without several other lymphoid populations/activation states to compare, e.g., naïve/activated T cells, ILCp (which might be truly naïve), and mature tissue ILC2s, which they suggest are recapitulated by IL-33 exposure. It would also perhaps be helpful to make these comparisons in mouse as this would provide more ready access to the different ILCs in different stages of development/tissue residency.
2. Li et al (Immunity. 2018 Feb 20; 48(2):258-270.e5.) showed that mouse ILC2s rely on VHL/HIF1a to regulate glycolytic/OXPHOS and IL-33 responsiveness; do similar pathways underlie the human ILC2s placed in hypoxic conditions?
3. Monticelli et al (Nat Immunol 17, 656–665;2016) showed two ILC2 populations

(CRTH2+ or ST2+) in human COPD and IPF patients and that ST2+ cells express arginase1 but not CRTH2+ cells. The heterogeneity in ST2 expression observed in this study (Supp. Fig. 3) also suggests that similar expression patterns among ILC2s may contribute to metabolic heterogeneity in the culture conditions –how do the authors account for this?
4. In Fig. 4, does modulation of glycolysis also affect production of other ILC2 factors (i.e., IL-5, IL-9, Areg). IL-9 in particular could act in an autocrine manner to enhance ILC2 survival.
5. It is unclear how viability/proliferation/cell numbers are assessed in the figures.
6. Do the MD patient ILC2s respond to cytokine or glycolysis interventions? Does metformin enhance ILC2 fitness or rescue poor fitness of ILC2 from MD patients due to its effect on ETC complex I?

Reviewer #3 (Remarks to the Author):

This paper describes a metabolic characterization of circulating human ILC2s, with experiments to link observed metabolic functions to cell survival, proliferation and cytokine production. It mixes analyses of cells immediately ex vivo and of cells cultured in vitro with IL-2 and IL-15, with or without IL-33. The take home message is that, compared to circulating NKdim cells, ILC2s have more mitochondria, and more mitochondrial membrane potential.

The authors chose to base the first half of their study on a comparison of ILC2s with NKdim cells, and most of the wow factor here comes from the finding that there is more mitochondrial mass, more mitochondrial fusion, and more mitochondrial membrane potential in the ILC2s than in the NKdim cells, but it is not clear how valid this comparison is. It would help if the authors would justify this focus more rigorously.

In general, the paper would be improved through the addition of a few more sentences in the main text to describe the basis for statements about upregulation, or increases or decreases of measured parameters (e.g. Figs 2A, S2d, 4A).

In the second part of the paper the emphasis is on the effects of IL-2/IL-7 and IL-33 on ILC2 metabolism. I find this more compelling. Here there is a clear effect of IL-33 on mitochondria and OXPHOS, linked to increased IL-13 production. Moreover, the data show a decline in cell numbers and in the expression of ST2 and GATA3 in low oxygen compared to higher oxygen concentrations in cells cultured in IL-2/7/33 or IL-2/7 alone, supporting the view that OXPHOS is critical for function. The addition of IL-33 was also shown to cause a marked increase in maximal glycolytic capacity.

In Fig. 3, it is not clear whether decreases in GATA3 and ST2 expression at low oxygen concentrations reflect a specific effect on the ILC2 program, or rather a broader effect on cell biology that encompasses a broad range of proteins, or only ILC2-lineage specific marks. It is also not clear whether these changes in expression reflect transcriptional or post-transcriptional effects. Effects of metabolic pathways on the regulation of transcription and translation is an area of considerable current interest, so some insights here would be valuable.

The authors show that OXPHOS, fueled by glutamine, is important for ILC2 survival, and claim that glycolysis is critical for IL-33-induced effector functions such as the production

of IL-33. While, based on the data, it is true that IL-3 production but not cell number/survival is affected by 2DG, there is also an effect of Rotenone and Antimycin A on IL-13 production that is at least equivalent to that of 2DG. The authors should untangle the significance of these observations. In this regard the paper would be greatly improved by using mass spec to trace the fate of carbon, etc., from the important fuels. In this way we would get an idea about why the various fuels are important for the cells under different conditions.

There is an over-reliance on inhibitors in this study. In Fig. 1 the authors show data from patients with mitochondrial diseases, and it would be great to know whether cells from these patients behave similarly to ILC2s from healthy donors after culture in IL-2/IL-7 plus or minus IL-33, as this would go some way towards rectifying this problem. We also need to know more about these patients – the precise description of their disease (s).

Author Rebuttal to Initial comments

Reviewers' comments:

Reviewer #1 (Remarks to the Author):

In this manuscript the Di Santo group investigated the metabolic requirements of human group 2 innate lymphoid cell (ILC2). Data presented by the authors here suggest that circulating “naïve” ILC2 have a higher level of oxidative phosphorylation (OXPHOS) than other ILC such as NK cell. Interestingly, ILC2 are severely reduced in patients with mitochondrial disease and impaired OXPHOS. Metabolomic and nutrient receptors analysis further demonstrated that ILC2 take up amino acids to sustain OXPHOS at steady-state. Upon activation with IL-33, ILC2 become highly proliferative relying on glycolysis and mTOR to produce IL-13, while continuing to fuel OXPHOS with amino acids to maintain cellular fitness and proliferation. Based on these observations the authors suggest that proliferation and function are metabolically uncoupled in human ILC2, offering new strategies to target ILC2 in disease settings.

The manuscript by Di Santo and colleagues is well put together and address key questions in the field of ILC2 biology. It is a refreshing read performed with human ILC2 and I congratulate the authors for this nice piece of work. However, while the presented set of data is very intriguing, essential controls are lacking and a big portion of claims are poorly justified by the presented data in its current form. Below major and some more specific comments are listed to support the authors to bring that manuscript to fruition and publication.

We thank the reviewer for these supportive comments and we appreciate the constructive criticism and comments that we have addressed to improve our manuscript.

Major comments

1. The authors claim that their presented data allows the conclusion that proliferation and function are metabolically uncoupled in human ILC2. This reviewer respectfully disagrees as single cytokine experimental controls are lacking throughout the manuscript. Throughout the manuscript the authors rely on the sole comparison of IL2+IL-7+IL-33 and IL-2+IL-7. Single cytokine stimulations need to be performed to distinguish the effects of IL-2 from IL-7, but most importantly to compare IL-33 only vs IL-33+IL-2, IL-33+IL-7 or IL-33+IL-2+IL-7.

We thank the reviewer for this comment, and we agree that analyses with individual cytokines are important to understand the essential roles of IL-2, IL-7 and IL-33 in the observed effects presented in the manuscript. We chose to compare the effects of IL2 + IL-7 with IL-2, IL-7 + IL-33 since previous studies had shown that circulating ILC2 are able to respond to these cytokines in vitro with robust expansion (Mjosberg, Nature Immunology, 2011; Bartemes, J Allergy Clin Immunol, 2014; Lim, JEM, 2016).
Consistent with these published results, we found that only combinations of IL-2 + IL7 or IL-2, IL-7 + IL-33 were able to maintain mitochondrial polarization and elevated TMRM levels when freshly isolated blood ILC2 were cultured in vitro in different cytokine combinations (Figure 1A for reviewer). Neither IL-2, IL-7 or IL-33 alone, or the combination IL-2-33 and IL-7-33, were able to maintain mitochondrial mass or polarization, or were able to expand blood ILC2 to any significant extent (Figure 1B for reviewer). This is partly due to the fact that blood ILC2 express very low levels of IL33 receptor (Supplemental Figure 3A). IL-33 alone was unable to support blood ILC2 in any fashion (Figure 1 A, B for reviewer). We have included these additional controls in the supplemental information (Supplemental Figures 4 D, E) and have discussed these results in the revised manuscript (lines 178-180, page 6).

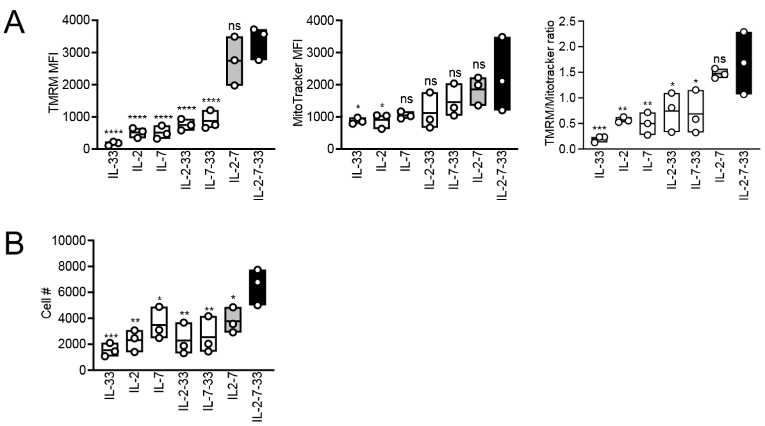

Figure 1 for reviewer

A) ILC2 were sorted from 3 healthy donors and cultured for 24 h in the indicated cytokines. Cells were stained with TMRM and MitoTracker and analyzed by FACS. B) FACS sorted ILC2 from 3 healthy donors were cultured for 5 days in the indicated cytokine combinations. Cell number was analyzed by FACS. Data representative of 2 independent experiments with 3 donors each. Statistics were assessed by one-way ANOVA with Dunnett correction, multiple comparison against IL-2-7-33; not significant (ns), *p<0.05; **p<0.01; ***p<0.001; ****p<0.0001.

2. When major experiments involve addition of various metabolic inhibitors the authors the show side-by-side, intracellular cytokine production, # of cells, proliferation as well as cell death. Do alterations of metabolic pathways lead to either, changes in cytokine production, altered proliferation, and/or cell death? These questions especially need to be addressed when altering oxygen availability (e.g. Figure 3E).

We have assessed the importance of different metabolic pathways and oxygen for ILC2 survival, proliferation and effector function. It is difficult to formally assess whether effects are linked (eg: whether decreased survival is the cause of poor cytokine production) since we can not easily enforce survival or proliferation in the human system. We found that upon IL-2-7 and IL-2-7-33, inhibition of amino acid metabolism (BCAT and BPTES) impacted mitochondrial fitness and proliferation but not function. Upon IL-33, only inhibition of glycolysis (by 2-DG) and OXPHOS (by RO & AA) led simultaneously to a decrease in ILC2 numbers and function. These findings suggest that ILC2 acquire a certain degree of flexibility in nutrient uptake in order to maintain mitochondrial fitness and concomitantly support proliferation and function. Consistent with data on OXPHOS inhibition, incubation under reduced oxygen conditions led to a reduction in ILC2 viability. What was not clear was how hypoxia impacts ILC2 function. We further explored this question and found that hypoxia upregulates HIF-1a expression potentially via PKM (Figure 7 for reviewer). This mechanistic underpinning had been previously reported to downmodulate ST2

expression and reduce murine ILC2 responsiveness to IL-33, thereby directly impacting ILC2 maturation and function (Li, Immunity, 2018). Our results confirming a similar mechanism operating in human ILC2 is now included (Supplemental Figure 5B, C) and discussed in the revised manuscript (lines 196-201, page 7).

3. Throughout the manuscript the author mostly analyzed IL-13 levels as a readout for cytokine production. Have the authors looked at other ILC2-producing cytokines such as IL-5, IL-6, and GM-CSF?

We thank the reviewer for this comment. ILC2 have been shown to express several 'type 2' cytokines, including IL-4, IL-5, IL-13 and GM-CSF (Salimi, JI, 2016; Fallone, JEM, 2016; Neill, Nature, 2010; Price, PNAS, 2010; Mjosberg, Nature Immunology, 2011). Amongst these, IL-5 is frequently studied in ILC2 and generally parallels IL-13 activity (Neill, Nature,2010; Price, PNAS, 2010; Mjosberg, Nature Immunology, 2011, Moro Nature, 2010; Molofsky, JEM, 2013). As requested by the reviewer, we studied IL-5 levels ir our system. In agreement with previous publications, we found that IL-5 levels followed the same trend as IL-13 (Figures 2 A, B for reviewer). We have included these data (Supplementa Figure 7B) and discussed these results in the revised manuscript (lines 247-250, page 8).

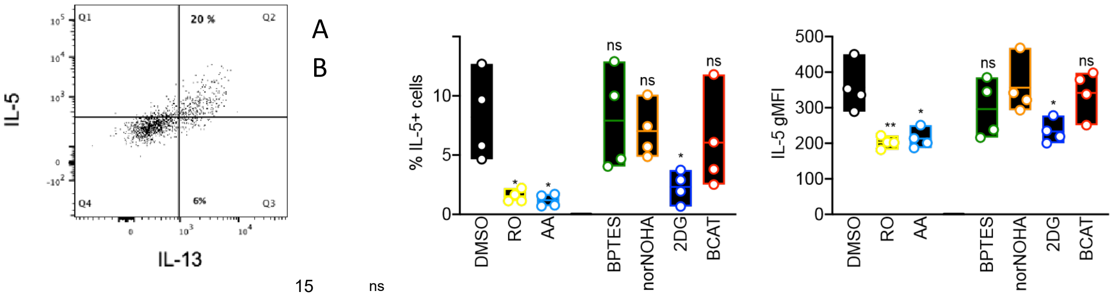

Figure 2 for reviewer

A, B) ILC2 were expanded in IL-2-7 for 7 days and then IL-33 was added for the last 6 hours. IL-5 and IL-13 were monitored by FACS. Data are summarizing 2 experiments with 3 donors each. Representative of a total of 3 independent experiments. Bars are ± s.e.m. Statistics were assessed by one-way ANOVA with Dunnett correction, not significant (ns), p>0.05; *p<0.05; **p<0.01; ***p<0.001.

In addition, has the production of anti-inflammatory and tissue restorative cytokines including IL-10 and Areg been explored? It will be important to test this to highlight of whether inflammatory as well as anti-inflammatory mediators have similar or distinct metabolic requirements.

We thank the reviewer for suggesting this experiment. As requested by the reviewer, we analyzed amphiregulin expression in ILC2 and assessed the metabolic requirements for its expression. We found that amphiregulin expression in human ILC2 was less affected by metabolic inhibitors (Figure 3 for reviewer). This suggests that inflammatory and anti-inflammatory mediators may have distinc t metabolic requirements. We have included these data (Supplemental Figure 7C) and discussed these results in the revised manuscript (lines 247-250, page 8).

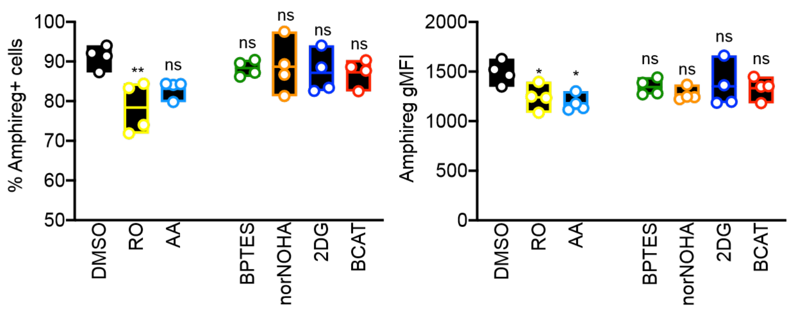

Figure 3 for reviewer

ILC2 were expanded in IL-2-7 for 7 days and then IL-33 was added for the last 6 hours. Amphiregulin was monitored by FACS. Data are summarizing 2 experiments with 3 donors each. Representative of a total of 3 independent experiments. Bars are ± s.e.m. Statistics were assessed by one-way ANOVA with Dunnett correction, not significant (ns), $p>0.05$; *$p<0.05$; **$p<0.01$; ***$p<0.001$.

4. Since this is a human study using patients as well as healthy donors, a table showing the clinicopathological data is warranted. Have the authors stratified patients by age, sex, and ethnic background (although it can be assumed that the patients and healthy donors are predominantly Caucasians) to determine if there are any differences seen in ILC2 levels?

We have included a table with clinicopathological data as requested (Supplemental Table 1) The levels of ILC2 in our control samples falls within the limits of those found in normal healthy individuals of European descent (Patin et al Nature Immunology, 2018) and the patients studied are of European descent.

5. The authors state that ILC2s are severely reduced in “Mitochondrial Disease”.

Mitochondrial Disease is an umbrella term encompassing various diseases such as Mitochondrial Myopathy and MERRF Syndrome, …… Can the authors show a breakdown of patients with the various diseases and to possibly stratify patients according to disease type and specific gene alteration to determine any differences.

We have included a table with a breakdown of patient disease subtypes and specific gene alterations as requested (Supplemental Table 1).

6. At the end of their manuscript the authors put forward the sweeping claim that "FA do not sustain bioenergetic needs but might accumulate upon cell division". This reviewer does not think that the authors present supportive evidence for this sweeping claim. (i) Although ETO treatment presented in data presented in Ext Fig. 4e does not show a statistically significant difference it could depend on does and kinetic of ETO treatment; (ii) FA oxidation could rely on peroxisomes rather than mitochondria; (iii) FA uptake could be decoupled from FA accumulation as liponeogenesis pathways could play a role here as well. (iv) Experiments restricting Glucose and or FA concentration in media would address this angle here.

As such I recommend the authors to discuss these aspects more carefully.

We thank the reviewer for these important comments. We have revised our conclusions concerning bioenergetic needs in human ILC2 taking the reviewer's constructive analyses into account. The relevant section of the revised manuscript can be found at lines 227-229, page 8.

7. Do the authors think that blood ILC2 behave metabolically similar to tissue ILC2? Can this aspect be at least touched on upon experimentally or discussed?

We thank the reviewer for suggesting a comparative experiment between blood and tissue ILC2. As requested, we made additional experiments to assess phenotype and metabolic properties of tissue ILC2 using tonsil samples. While ILC2 are somewhat scarce in these secondary lymphoid tissues (Figure 4A for reviewer), they are an easily accessible human sample. Moreover, previous studies have reported that tonsillar ILC2 show conserved ILC2 functional profiles in terms of transcription factor expression and cytokine production (Mazzurana, Cell Res, 2021).

We confirmed that tonsillar ILC2 have high GATA-3 expression (Figure 4B for reviewer) and a similar CD127+CD161+ phenotype with scant IL-33R expression (Figure 4C for reviewer). CRTh2 expression was down-regulated in tonsil ILC2 (Figure 4D for reviewer) consistent with previous observations (Mazzurana, Cell Res, 2021) · Concerning metabolic parameters, we did not find remarkable differences between blood and tonsil ILC2, suggesting that many of these tissue ILC2 may be blood-derived and not (yet) activated by local environmental signals (Figure 4E-F for reviewer) · Tonsil ILC2 retain highly polarized mitochondria (Figure 4E for reviewer) and trend towards higher CD36 levels (Figure 4F for reviewer). We have included these new results as Supplemental Figures 1G-K and 3E in the revised manuscript (lines 111-117, pages 4 and lines 151-152, page 5).

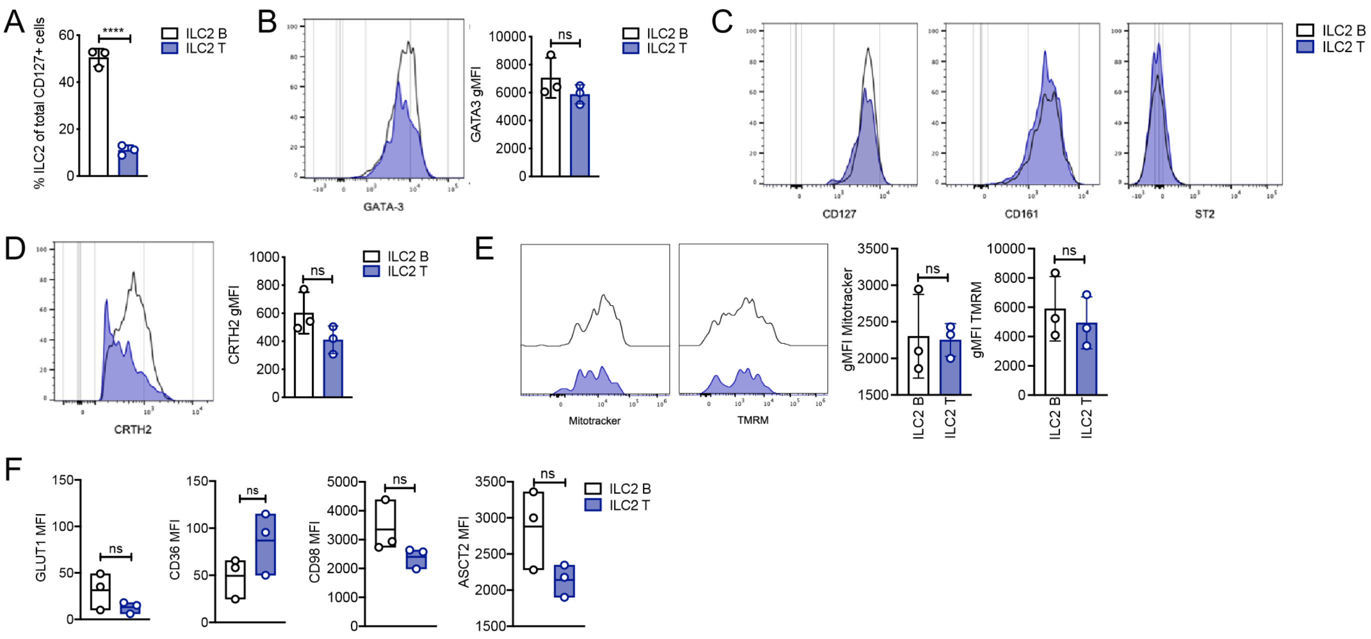

Figure 4 for reviewer

A-E) Pediatric paired blood and tonsil from 3 donors were analyzed by FACS for the indicate markers: A) Percentage of ILC2, B) GATA-3, C) CD127, CD161, ST2, D) CRTH2 and E) TMRM and Mitotracker. F) FACS analysis of nutrient receptor analysis: GLUT1, CD36, CD98 and ASCT2. Data are representative of 2 independent experiments with 3 donors each. Bars are ± s.e.m. Statistics were assessed by t-test, not significant (ns), ****p<0.0001.

Specific comments

1. Either table 1 was lacking or was mislabelled as a Figure, but this reviewer did not have access to data referred to as Table 1.

We apologize for this omission. The relevant information is now provided as Table 2.

2. Figure 1B: Frequencies need to be supplemented by absolute cell counts to allow conclusions made by the authors (e.g. number of ILC2 in relation to total CD45positve cells).

Unfortunately, we cannot provide absolute cell counts because MD donors were processed by collaborators. MD have generally normal blood counts (leukocytes are the normal range). This information is now included in Table 1.

3. Fig 1 showed flow cytometry plots showing Mitotracker and TMRM and looking at ILC2s vs NKDim. Unstained Control peaks should be included.

We include unstained controls are requested.

4. ILC2 gating strategy (Supp Fig 1A) showed CD94-NKG2A-, although the supposed double negative population looks are and entering the 103 scale. It might help the authors to extend the scales and provide a FMO to ensure proper gating.

In this supplemental figure, ILC2 are correctly pre-gated as Lin-CD7+CRTh2+ cells · As NK cells are negative for CRTh2, the CD94/NKG2A expression on the gated ILC2 can be considered as background. We include our staining of CD94/NKG2A expression on NK cells below for comparison.

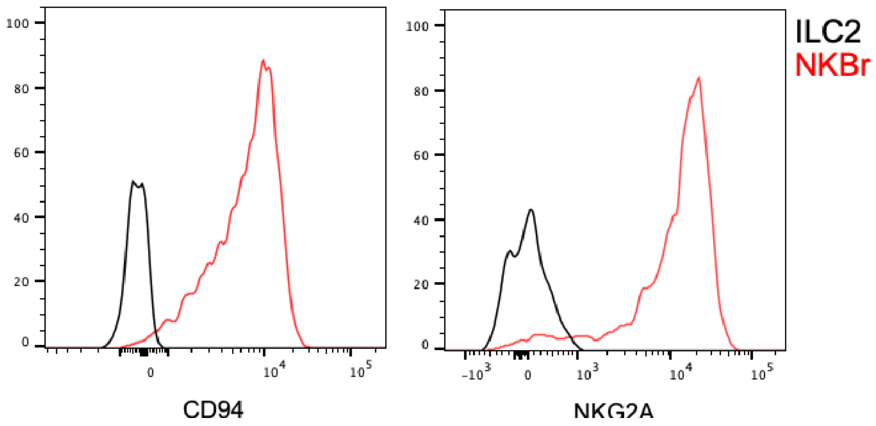

5. Fig 1 showed MFI. Was it mean or median? While knowing that the peaks are normally distributed is an important factor for MFI, perhaps it might help the authors to use Geometric MFI (gMFI) for their analysis?

Geometric MFI was used in the analysis across all the manuscript. We include "gMFI" in all the plots and figure legends.

6. Fig 1E looks like the NKDim and ILC2s are acquired on separate events, or could it be a compensation issue since the double negative populatons look different between the 2 cell types.

These populations were sorted separately using different gating strategies (as indicated in the figure legends and methods) .

7. Fig 2b the authors performed RNASeq on 3 healthy donors. It might help the authors to do RNASeq on the patient blood and provide a heatmap looking at different gene expression?

We thank the reviewer for this suggestion. Unfortunately, it is not possible to perform RNA sequencing on the patient samples due to very low ILC2 numbers in these patients .

Do these transporters change after combined activation with IL2/IL-7+IL-33? It would be important to shed light on this? Can RNA-Seq data be complemented by flow cytometric data of some of the highest expression transporters?

We included FACS analysis (protein expression) of several nutrient transporters that could be detected by antibodies or other methods (viral envelope interactions; Manel, Cell, 2013; Oburoglu, Cell Stem Cell, 2014) following cytokine stimulation (original Figure 4b).

8. Fig 2 the authors used nor-NOHA, BCATi, BPTES, and 2DG at 1μM. How did the authors determine optimal concentrations? Can they provide a titration curve, or a dose-response curve to explain why 1μM was used?

We performed titration experiments for inhibitors in ILC2 cultured with IL-2-7 and IL-2-7-33 and assessed effects on cell number and viability (annexin V staining). We could observe detrimental effects on cell viability at 10uM, especially in the case of BPTES and BCAT, so 1uM was used as a final dose for our experiments (Figure 5 for reviewer).

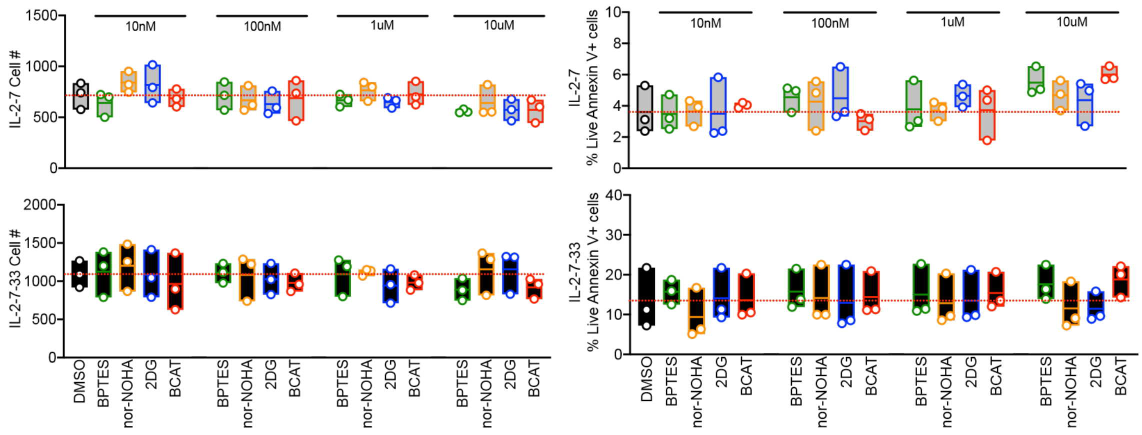

Figure 5 for reviewer

FACS sorted ILC2 were expanded for 7 days in IL-2-7 and then IL-33 was added or not for 72h. In the last 18h inhibitors (BPTES, 2DG, nor-NOHA, BCATi) were added or not at the indicated concentrations. Cell number and Annexin V were analyzed by FACS. Data representing of 3 independent experiments with 2 to 3 donors each. Bars are ± s.e.m.

9. Fig 3A the authors state that cell counts were determined by FACS. Was it by counting beads, or just by regular gatings? Will trypan blue and a haemocytometer validate those results? Please give details in the methods sections.

Cell number was determined either by FACS (based on gating: singlets, live cells) or trypan blue. This information has been added in the methods.

10. Fig 3 showed expansion of ILC2s for 7 days with IL-2 + IL-7 and then stimulated with or without IL-33 for 72 hours. Did the authors do any cytokine or serum starvation prior to IL-33 stimulation, to ensure the cells were back to baseline levels?

In our study, we aimed to understand the metabolic transitions associated with ILC2 priming and activation. Assessing the impact of cytokine or serum starvation would address a different question that is not necessarily pertinent in this context. In addition, cytokine or serum starvation could result in metabolic reprogramming confounding the interpretation.

11. Fig 3E x-axis label to include Oxygen level

The figure has been modified accordingly.

12. Fig 3E, have the authors looked at other ILC2 activation and co-stimulatory markers such as ICOS, KLRG1, Thy1, and CD25 or CD127?

We have assessed expression of CD161, ICOS, KLRG1 as well as CD45, CD25, CD127, CD7 and TSLPR on ILC2 following cytokine activation in normoxia and hypoxia as requested by reviewer #3 (see below). These new results are included (Supplemental Figure 5A) in the revised manuscript.

13. Figs 4D and 4E no stats for D6?

Statistical analyses are included in all figures.

Reviewer #2 (Remarks to the Author):

The manuscript by Surace et al. presents a metabolic characterization of human circulating ILCs, using freshly sorted ILC2s and CD56+CD16+NKDim cells, as well as ILC2s exposed to various cytokines in vitro. The authors show that circulating ILC2s have relatively enhanced mitochondrial function as compared to NK cells. In peripheral blood of patients with mitochondrial disorders, the frequency of ILC2s are reduced, and exhibit lowered mitochondrial mass/polarization, decreased GATA-3 staining and increased Annexin-V staining, suggesting that ILC2 function and survival are impaired in the setting of MD. In support of this, inhibition of ETC complex I and III in cultured ILC2s impaired survival, whereas NKDim cells were unaffected. Metabolomic analysis indicated an enrichment of arginine and branched amino acids (BCAA) in ILC2s along with the membrane transporters LAT1 and LAT4, while inhibition of arginase-1 and BCAT pathways reduced mitochondria and OXPHOS and altered corresponding transcriptional pathways in ILC2s. Culturing sorted ILC2s with IL-2/7/33 increased OCR and DSRC compared to IL-2/7 alone, and inhibition of ETC complex I and III impaired ILC2 survival. The authors suggest that ILC2s maintain both OXPHOS and enhanced glycolytic pathways during activation to maintain both cell fitness and IL-13 production, respectively. This manuscript is clear and concisely written, adding an important analysis of human ILC2 metabolism to the field.

We thank the reviewer for their positive comments on our manuscript.

The observations are somewhat limited in this brief report; however, particularly with respect to issues of development/tissue residency that are raised in the text.

Specifically:

1. The authors draw attention to the unique and ‘non-naïve’ metabolic profile of circulating ILC2s, but compare only to NKDim cells, which they previously compared with NKbright cells in a recent publication describing similar relative differences (Blood Adv (2021) 5 (1): 26–38). It is difficult to get a sense of what makes the ILC2 metabolic profile unique without several other lymphoid populations/activation states to compare, e.g., naïve/activated T cells, ILCp (which might be truly naïve), and mature tissue ILC2s, which they suggest are recapitulated by IL-33 exposure. It would also perhaps be helpful to make these comparisons in mouse as this would

provide more ready access to the different ILCs in different stages of development/tissue residency.

We thank the reviewer for this suggestion. We have analyzed key mitochondrial characteristics in blood ILC2 compared with other circulating immune cell subsets. We found that ILC2 have a unique phenotype compared to circulating naïve and central/effector memory CD4 T cells (Figure 6 for reviewer). This unusual mitochondrial signature suggests that circulating ILC2 may have already be exposed to distinct environmental signals, perhaps including those emanating from their passage through tissues. We include this new results in the revised manuscript as
Supplemental Figure 1F (lines 105-109, page 4).

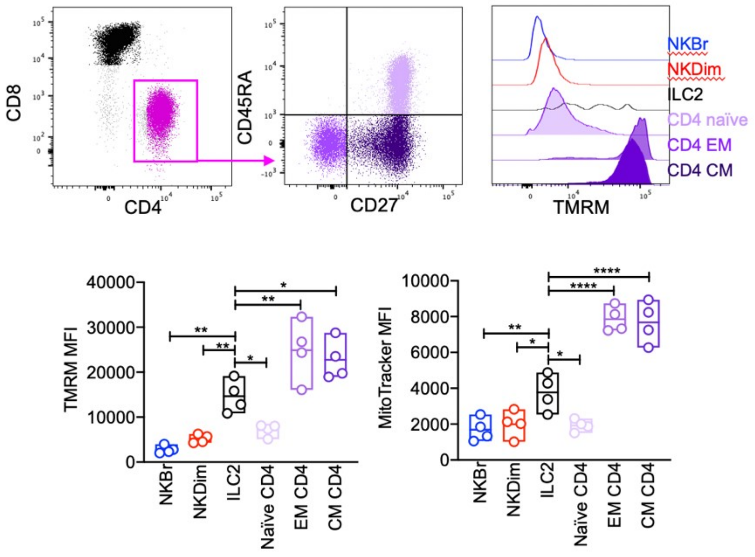

Figure 6 for reviewer

TMRM and MitoTracker measurement in CD4 naïve, effector memory (EM), central memory (CM), NK and ILC2 from the blood of healthy donors. Data representative of 2 experiments with at least 3 donors each. Statistics were assessed by one-way ANOVA with Dunnett correction, not significant (ns), $p>0.05$; *$p<0.05$; **$p<0.01$; ****$p<0.0001$.

2. Li et al (Immunity. 2018 Feb 20; 48(2):258-270.e5.) showed that mouse ILC2s rely on VHL/HIF1a to regulate glycolytic/OXPHOS and IL-33 responsiveness; do similar pathways underlie the human ILC2s placed in hypoxic conditions?

We thank the reviewer for this suggestion. We analyzed the effects of hypoxia on HIF1a expression and found that hypoxia increased HIF1a (Figure 7A for reviewer) with a concomitant decrease in ST2 expression (Figure 3G of the revised manuscript) in IL-2-7-33 stimulated human ILC2 consistent with the results of Li et al. We further examined the proposed mechanistic underpinnings of this effect (PKM up-regulation) and found that hypoxia dramatically up-regulated PKM expression in IL-33-stimulated human ILC2 (Figure 7B for reviewer). These observations suggest that a conserved pathway coupling HIF1a to PKM operates in human ILC2 when oxygen levels are limiting. We include this new results in the revised manuscript as Supplemental Figure 5B, C (lines 197-201, page 7).

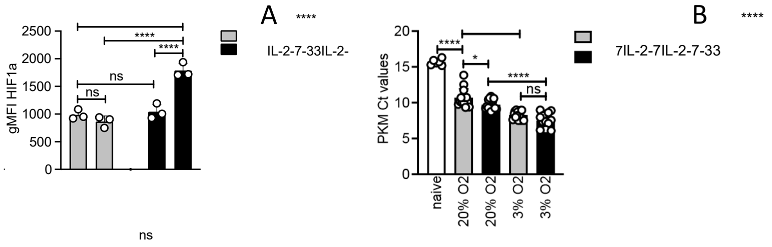

Figure 7 for reviewer

A-B) Cells were cultured at 20% or 3% oxygen in IL-2-7 or IL-2-7-33 for 5 days. A) Cell count, HIF-a, ST2 and GATA-3 expression were analyzed by FACS. B) On day 5, ILC2 were sorted in small bulks and the transcript levels of PKM were analyzed by Biomark. A) Data representative of 3 independent experiment with 3 donors each. B) Data pooled summarizing

2 experiments with 3 donors each. Bars are ± s.e.m. Statistics were assessed by one-way ANOVA with Dunnett correction, not significant (ns), $p>0.05$; *$p<0.05$; **$p<0.01$; ***$p<0.001$; ****$p<0.0001$.

3. Monticelli et al (Nat Immunol 17, 656–665;2016) showed two ILC2 populations (CRTH2+ or ST2+) in human COPD and IPF patients and that ST2+ cells express arginase1 but not CRTH2+ cells. The heterogeneity in ST2 expression observed in this study (Supp. Fig. 3) also suggests that similar expression patterns among ILC2s may contribute to metabolic heterogeneity in the culture conditions – how do the authors account for this?

We thank the reviewer for this comment. The previous Monticelli et al. study used samples from patients with chronic pulmonary disorders for which ILC2 might differ from those found in healthy individuals. We analyzed CRTh2 and ST2 expression on blood ILC2 and found that are all blood ILC2 are CRTh2 positive and ST2 negative, suggesting limited heterogeneity for these markers in human healthy donors (Figure 8A for reviewer). In contrast, upon stimulation with IL-2 and IL-7, roughly 50% of blood ILC2 become ST2+ by day 5 and by day 8, the majority of cytokine stimulated ILC2 are ST2+ (around 90%) (Figure 8B for reviewer). These results suggest cytokines can positively regulate ST2 expression and in the frame of our experimental design, would allow the majority of the IL-2 and IL-7 stimulated ILC2 to respond to IL-33. We include this new results in the revised manuscript as Supplemental Figure 4A, C (lines 176-178, page 6).

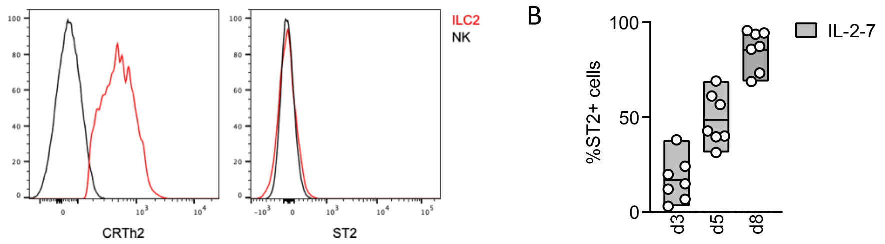

A

Figure 8 for reviewer

A) Representative plot of CRTh2 and ST2 expression in ILC2 and total NK cells in blood of healthy donors. B) Sorted ILC2 were cultured with IL-2-7 for the indicated days. ST2 expression was monitored by FACS. A) Data representative of 4 experiments with 3 to 4 donors each. B) Data pooled summarizing 3 independent experiments with at least 2 donors.

4. In Fig. 4, does modulation of glycolysis also affect production of other ILC2 factors

(i.e., IL-5, IL-9, Areg). IL-9 in particular could act in an autocrine manner to enhance ILC2 survival.

We thank the reviewer for this comment. We analyzed effects of glycolysis inhibitors on IL-5 and amphiregulin production by cultured ILC2 (please see response to Reviewer #1, comment 3). We found that glycolysis inhibited IL-5 production (similar to that observed for IL-13). In contrast, amphiregulin production was not affected suggesting distinct metabolic regulation of ILC2-dependent production of soluble immune mediators. We have included these data Supplemental Figures 7B, C and discussed these results in the revised manuscript (lines 247-250, page 8).

5. It is unclear how viability/proliferation/cell numbers are assessed in the figures.

We apologize for any confusion. We have clarified the methods and figures legends to indicate how viability, proliferation and cell numbers were assessed.

6. Do the MD patient ILC2s respond to cytokine or glycolysis interventions? Does metformin enhance ILC2 fitness or rescue poor fitness of ILC2 from MD patients due to its effect on ETC complex I?

Unfortunately, we can not perform these proposed studies due to the very limited cell numbers in the MD patients.

Reviewer #3 (Remarks to the Author):

This paper describes a metabolic characterization of circulating human ILC2s, with experiments to link observed metabolic functions to cell survival, proliferation and cytokine production. It mixes analyses of cells immediately ex vivo and of cells cultured in vitro with IL-2 and IL-15, with or without IL-33. The take home message is that, compared to circulating NKdim cells, ILC2s have more mitochondria, and more mitochondrial membrane potential.

The authors chose to base the first half of their study on a comparison of ILC2s with NKdim cells, and most of the wow factor here comes from the finding that there is more mitochondrial mass, more mitochondrial fusion, and more mitochondrial membrane potential in the ILC2s than in the NKdim cells, but it is not clear how valid this comparison is. It would help if the authors would justify this focus more rigorously.

In general, the paper would be improved through the addition of a few more sentences in the main text to describe the basis for statements about upregulation, or increases or decreases of measured parameters (e.g. Figs 2A, S2d, 4A).

We thank the reviewer for their very constructive comments. As requested, we have included additional comments concerning our observed findings.

In the second part of the paper the emphasis is on the effects of IL-2/IL-7 and IL-33 on ILC2 metabolism. I find this more compelling. Here there is a clear effect of IL-33 on mitochondria and OXPHOS, linked to increased IL-13 production. Moreover, the data show a decline in cell numbers and in the expression of ST2 and GATA3 in low oxygen compared to higher oxygen concentrations in cells cultured in IL-2/7/33 or IL2/7 alone, supporting the view that OXPHOS is critical for function. The addition of IL-33 was also shown to cause a marked increase in maximal glycolytic capacity.

In Fig. 3, it is not clear whether decreases in GATA3 and ST2 expression at low oxygen concentrations reflect a specific effect on the ILC2 program, or rather a broader effect on cell biology that encompasses a broad range of proteins, or only ILC2-lineage specific marks. It is also not clear whether these changes in expression reflect transcriptional or post-transcriptional effects. Effects of metabolic pathways on the regulation of transcription and translation is an area of considerable current interest, so some insights here would be valuable.

We thank the reviewer for this interesting comment regarding the effects of hypoxia on ILC2 phenotype and function. We examined whether incubation of ILC2 in low oxygen conditions selectively affected the ILC2 program or more broadly affected lineage non-specific markers. In particular, we assessed expression of several wellestablished ILC2-related proteins in ILC2 cultured with cytokines under normoxia and hypoxia. These markers included CD161, GATA-3, ICOS, KLRG1 and ST2. In addition, we analyzed other lymphocyte markers including CD45, CD25, CD127, CD7 and TSLPR. We found evidence for selective effects on ST2, GATA3 and to a lesser extent on CD161 (Figure 9A for reviewer). In contrast, other ILC2 markers (KLRG1, ICOS) were unaffected as were markers not restricted to ILC2 (CD45, CD127, CD25, CD7, TSLPR). These results suggest that hypoxia regulates a specific ILC2 subprogram that centers around GATA-3 and ST2 expression and is not globally affecting cell biology in a non-specific fashion. We have included these data Supplemental Figure 5A and discussed these results in the revised manuscript (lines 194-196, pages 7).

Concerning transcriptional regulation of affected gene targets, we analyzed the effects of hypoxia on mRNA expression of GATA-3 and ST2 (Figure 9B for the reviewer). We found decreased mRNA levels for these two ILC2-associated target genes suggesting that hypoxia regulates their transcriptional activity. We have included these data in Supplemental Figure 5C and discussed these results in the revised manuscript (lines 197-201, pages 7).

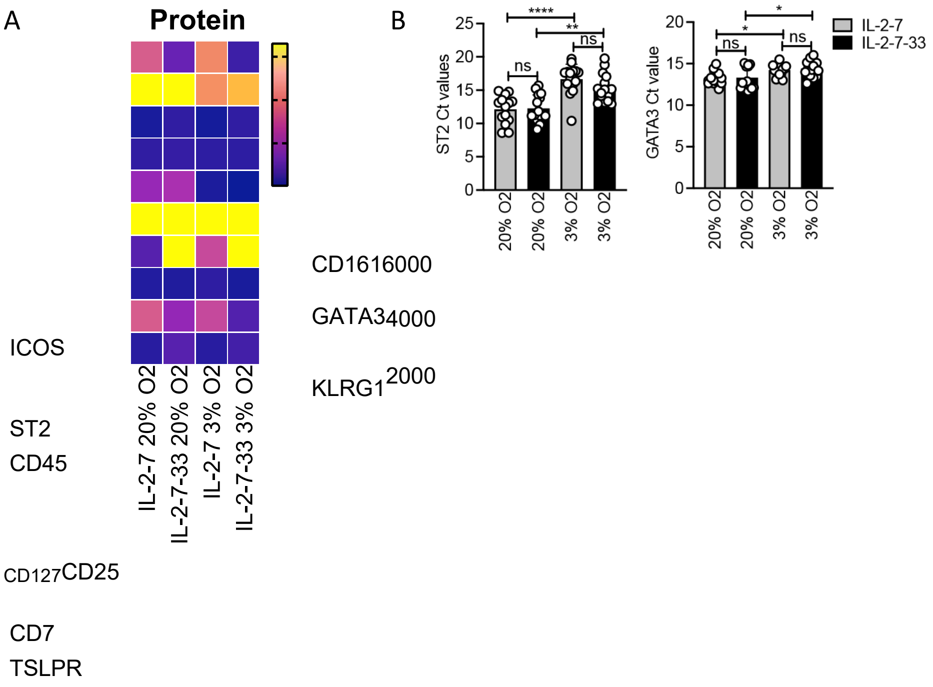

Figure 9 for reviewer

A-B) Cells were cultured at 20% or 3% oxygen in IL-2-7 or IL-2-7-33 for 5 days. A) Expression of the indicated markers was analyzed by FACS (gMFI). B) On day 5, ILC2 were sorted in small bulks and the transcript levels of ST2 and GATA3 were analyzed by Biomark. Data pooled summarizing A) 3 independent experiments with 3 donors each, B) 2 independent experiment with 3 donors each. Bars are ± s.e.m. Statistics were assessed by one-way ANOVA with Dunnett correction, not significant (ns), $p>0.05$; *$p<0.05$; **$p<0.01$; ****$p<0.0001$.

The authors show that OXPHOS, fueled by glutamine, is important for ILC2 survival, and claim that glycolysis is critical for IL-33-induced effector functions such as the production of IL-33. While, based on the data, it is true that IL-3 production but not cell number/survival is affected by 2DG, there is also an effect of Rotenone and Antimycin A on IL-13 production that is at least equivalent to that of 2DG. The authors should untangle the significance of these observations. In this regard the paper would be greatly improved by using mass spec to trace the fate of carbon, etc., from the important fuels. In this way we would get an idea about why the various fuels are important for the cells under different conditions.

We thank the reviewer for these interesting suggestions. We understand the interest in tracking carbon fates using labeled nutrients, however, we would propose that these experiments be the subject of a future report. Concerning the regulation of IL-13 production, it has been shown that glucose impacts on lymphocytes function and proliferation through mTOR, an essential nutrient sensor (Cheng, Science, 2014; Karagiannis, Immunity 2020). Moreover, ROS production plays a role in mouse ILC activation (C Zheng, Cell Mol Immunol, 2020; Di Luccia, J Exp Med, 2019). We found that IL-33 activation induces ROS production in human ILC2. Treatment of IL-33activated ILC2 by the ROS scavenger MitoTempo or by the mTOR inhibitor rapamycin further confirmed the importance of these pathways for IL-13 production (Figure 10 for the reviewer). We have included these data in Supplemental Figure 7E, F and discussed these results in the revised manuscript (lines 252-259, pages 8-9).

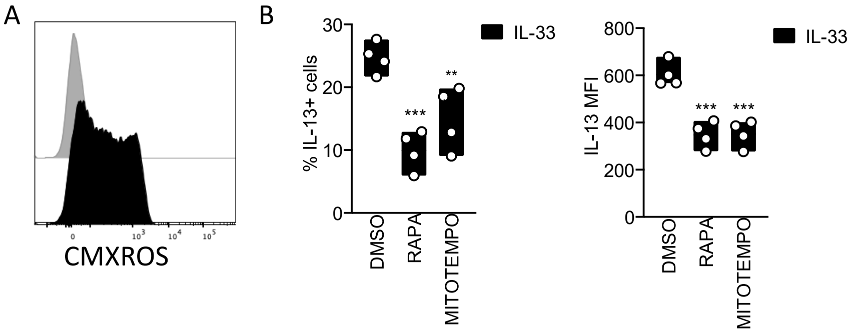

Figure 10 for reviewers

A) ILC2 were expanded for 7 days in IL-2-7 and then IL-33 was added or not for 72h. ROS staining was performed by FACS. B) ILC2 were expanded in IL-2-7 for 7 days in the presence of Rapamycin, MitoTempo or DMSO and then IL-33 was added for the last 6 hours on normal medium. IL-13 was monitored by FACS. Data from 2 independent experiments with at least 3 donors. Bars are ± s.e.m. Statistics were assessed by one-way ANOVA with Dunnett correction, **p<0.01; ***p<0.001.

There is an over-reliance on inhibitors in this study. In Fig. 1 the authors show data from patients with mitochondrial diseases, and it would be great to know whether cells from these patients behave similarly to ILC2s from healthy donors after culture in IL-2/IL-7 plus or minus IL-33, as this would go some way towards rectifying this problem. We also need to know more about these patients – the precise description of their disease (s).

Unfortunately, we can not perform these proposed studies due to the very limited cell numbers in the MD patients. However, we have added a table with clinical parameters for the patients used in this study.

Decision Letter, first revision:

Subject: Decision on Nature Immunology submission NI-LE31657A-Z
Message: 2nd Aug 2021

Dear Jim,

Thank you for providing your rebuttal to the referees' remaining comments on your manuscript entitled"Dichotomous metabolic networks govern human ILC2 proliferation and

function". I think your responses to the major concern posed by referees #2 and #3 about the heterogeneity of the TMRM staining is likely due to human donor-to-donor variability, thus I would encourage you to revise the manuscript with the inclusion of Referee figures 1 and 3, as shown in your point-by-point response. The other clarifications are fine. I would also suggest increasing the figure resolution of Fig. 1a,e. These images are not as crisp as others in the manuscript.

We therefore invite you to revise your manuscript taking into account all reviewer and editor comments. Please highlight all changes in the manuscript text file in Microsoft Word format.

We are committed to providing a fair and constructive peer-review process. Do not hesitate to contact us if there are specific requests from the reviewers that you believe are technically impossible or unlikely to yield a meaningful outcome.

When revising your manuscript:

* Include a “Response to referees” document detailing, point-by-point, how you addressed each referee comment. If no action was taken to address a point, you must provide a compelling argument. This response will be sent back to the referees along with the revised manuscript.

* If you have not done so already please begin to revise your manuscript so that it conforms to our Letter format instructions at http://www.nature.com/ni/authors/index.html. Refer also to any guidelines provided in this letter.

* Please include a revised version of any required reporting checklist. It will be available to referees to aid in their evaluation of the manuscript goes back for peer review. They are available here:

Reporting summary:
https://www.nature.com/documents/nr-reporting-summary.pdf

When submitting the revised version of your manuscript, please pay close attention to our href="https://www.nature.com/nature-research/editorial-policies/image-integrity">Digital Image Integrity Guidelines. and to the following points below:

-- that unprocessed scans are clearly labelled and match the gels and western blots presented in figures.
-- that control panels for gels and western blots are appropriately described as loading on sample processing controls
-- all images in the paper are checked for duplication of panels and for splicing of gel lanes.

Finally, please ensure that you retain unprocessed data and metadata files after publication, ideally archiving data in perpetuity, as these may be requested during the peer review and production process or after publication if any issues arise.

Please use the link below to submit your revised manuscript and related files:

[REDACTED]

Note: This URL links to your confidential home page and associated information about manuscripts you may have submitted, or that you are reviewing for us. If you wish to forward this email to co-authors, please delete the link to your homepage.

We hope to receive your revised manuscript within four weeks. If you cannot send it within this time, please let us know. We will be happy to consider your revision so long as nothing similar has been accepted for publication at Nature Immunology or published elsewhere.

Please do not hesitate to contact me if you have any questions or would like to discuss these revisions further.

Nature Immunology is committed to improving transparency in authorship. As part of our efforts in this direction, we are now requesting that all authors identified as 'corresponding author' on published papers create and link their Open Researcher and Contributor Identifier (ORCID) with their account on the Manuscript Tracking System (MTS), prior to acceptance. ORCID helps the scientific community achieve unambiguous attribution of all scholarly contributions. You can create and link your ORCID from the home page of the MTS by clicking on 'Modify my Springer Nature account'. For more information please visit please visit www.springernature.com/orcid.

We look forward to seeing the revised manuscript and thank you for the opportunity to review your work.

Kind regards,

Laurie

Laurie A. Dempsey, Ph.D.
Senior Editor
Nature Immunology
l.dempsey@us.nature.com
ORCID: 0000-0002-3304-796X

Reviewers' Comments:

Reviewer #1:
Remarks to the Author:
The authors addressed all queries and questions and substantially added information to their manuscript. The work is cohesive and well put together. I recommend to accept the manuscript at its current for.

Reviewer #2:
Remarks to the Author:
The authors have added new data in their responses to reviewers, but concerns remain about the major findings, as follows:

<i>(R1) We thank the reviewer for this suggestion. We have analyzed key mitochondrial characteristics in blood ILC2 compared with other circulating immune cell subsets. We found that ILC2 have a unique phenotype compared to circulating naïve and central/effector memory CD4 T cells (Figure 6 for reviewer). This unusual mitochondrial signature suggests that circulating ILC2 may have already be exposed to distinct environmental signals, perhaps including those emanating from their passage through tissues. We include this new results in the revised manuscript as Supplemental Figure 1F (lines 105-109, page 4).</i>

The new ILC2 data in S1F look quite different than the other ILC2 histograms showing TMRM (e.g., 1A), now indicating three or more peaks distributed along the axis that overlap with the NK/CD4 cell peaks; what is the basis for this variation? MFI would not seem to adequately represent what appears to be distinct hi/lo/mid ILC2 subsets, and new Mitotracker histogram data are not shown. Although the authors have added new comparisons with T cells, it remains difficult to gauge the relevance of the different ILC2 TMRM/Mitotracker measurements, which appear to be highly dependent on particular assay-to-assay conditions that are either not adequately controlled, or are variable enough from subject to subject to cause a significant concern about interpretation.
The authors suggest that the basis for metabolic 'uniqueness' of circulating ILC2s is differential transit through tissues or exposure to different environmental signals but do not address this experimentally. The new tonsil ILC2 data suggest that tissue residency in fact does not determine metabolic 'naivety' and comparisons with other tonsil ILC populations, particularly ILC precursors, are not shown. In addition, the lack of ST2 expression on blood/tonsil ILC2s would suggest that IL-33 does not influence metabolic changes in vivo.

<i>(R4). We thank the reviewer for this comment. We analyzed effects of glycolysis inhibitors on IL-5 and amphiregulin production by cultured ILC2 (please see response to Reviewer #1, comment 3). We found that glycolysis inhibited IL-5 production (similar to that observed for IL-13). In contrast, amphiregulin production was not affected suggesting distinct metabolic regulation of ILC2-dependent production of soluble immune mediators. We have included these data Supplemental Figures 7B, C and discussed these results in the revised manuscript (lines 247-250, page 8). </i>

Unfortunately the authors did not evaluate IL-9, which acts in an autocrine/paracrine manner to influence ILC2 survival and fitness, as well as production of IL-5, IL-13, and amphiregulin in different settings (Wilhelm et al., Nat Immunol. 2011 Oct 9;12(11):1071-7; Turner et al., J Exp Med. 2013 Dec 16;210(13):2951-65; Mohapatra et al., Mucosal Immunology 2016 9:275–286).

Reviewer #3:
Remarks to the Author:
The paper is improved by the revision, but I have a few questions based on the new emphasis on a few of the points addressed in the revision.

I have difficulty with the TMRM plot in Fig. S1F – there are multiple peaks for the ILC2s. This can also be seen in Figs. S1K and 3D. This implies that there is heterogeneity in the ILC2 population, with each peak representing a population of cells that has mitochondria with membrane potential that is distinct from the cells represented by the other peaks. I think that the authors are trying to say as much when they refer to cells having exposure to "distinct environmental signals" (page 4, line 110), but this description and related implications need to be more clearly stated in the text. Is there a precedent for this type of staining in other cell types? Have the authors attempted to gate on each of the peaks and probe for marks that discriminate between the distinct populations? This seems like an important point to me.

It is not clear to me why a similar TMRM staining pattern in tonsil pDC vs. blood pDC suggests that they are "not yet rewired metabolically by local ..signals" (line 118). Is the tissue-enforced metabolic rewiring that they are alluding to here the effects of IL-2, -7 and -33? If so, this should be explicitly stated.

It would be good to see whether any of the metabolic inhibitors that affect function result in a reduction in heterogeneity in the TMRM staining pattern - are there ILC2s , represented by peaks, that are preferentially affected by the manipulation of different metabolic pathways? I imagine that the authors have these data already, since they show data from TMRM measurements in e.g. Figs. 1F,G, 2F,G, but only as bar graphs.

Page 5, line 149. LAT1 is composed of SLC3A2/CD98 and SLC7A5, and expression of the latter is not elevated, so I don't think that it is appropriate to talk about increased expression of LAT1 without functional assays or evidence of increased SCL7A5 expression. SLC3A2 also partners other SLCs in other amino acid transporters.

Line 170. It is not clear what the authors are referring to when they say "metabolically naïve". Please expand discussion around this idea.

Text references to Figure 3 are muddled – the call outs in the text after 3d are incorrect.

Author Rebuttal, first revision:

Reviewer #1

(Remarks to the Author)

The authors addressed all queries and questions and substantially added information to their manuscript. The work is cohesive and well put together. I recommend to accept the manuscript at its current form.

We thank the reviewer for these positive comments on our revised manuscript.

Reviewer #2

(Remarks to the Author)
The authors have added new data in their responses to reviewers, but concerns remain about the major findings, as follows:

(R1) We thank the reviewer for this suggestion. We have analyzed key mitochondrial characteristics in blood ILC2 compared with other circulating immune cell subsets. We found that ILC2 have a unique phenotype compared to circulating naïve and central/effector memory CD4 T cells (Figure 6 for reviewer). This unusual mitochondrial signature suggests that circulating ILC2 may have already be exposed to distinct environmental signals, perhaps including those emanating from their passage through tissues. We include this new results in the revised manuscript as Supplemental Figure 1F (lines 105-109, page 4).

The new ILC2 data in S1F look quite different than the other ILC2 histograms showing TMRM (e.g., 1A), now indicating three or more peaks distributed along the axis that overlap with the NK/CD4 cell peaks; what is the basis for this variation? MFI would not seem to adequately represent what appears to be distinct hi/lo/mid ILC2 subsets, and new Mitotracker histogram data are not shown. Although the authors have added new comparisons with T cells, it remains difficult to gauge the relevance of the different ILC2 TMRM/Mitotracker measurements, which appear to be highly dependent on particular assay-to-assay conditions that are either not adequately controlled, or are variable enough from subject to subject to cause a significant concern about interpretation.

The results included in Supplemental Figure 1F in our revised manuscript were made in response to the reviewer's previous request that we compare mitochondrial characteristics (TMRM/Mitotracker staining) in blood ILC2 versus other circulating immune subsets. We apologize for not having included the Mitotracker histogram data. The reviewer raises concerns re: variability in the TMRM/Mitotracker measurements and the impact of such variability on interpretation of results. The observed heterogeneity in the staining (for example, comparing S1F to Fig 1A) is not the result of experimental variation, but rather reflects donor-to-donor variability. As it is the case for human cohorts, "healthy individuals" differ considerably in their characteristics, lifestyle, and clinical history. This heterogeneity in mitochondrial mass and polarization has been previously reported in $CD8^+$, $CD4^+$ and γδT cells (Sukumar M. et al, Cell Metabolism, 2016; Callender L.A. et al, Nat Comm, 2021; Lopes N., Nat Imm, 2021) and we recently demonstrated this in NKDim cells of healthy individuals (Surace L. et al, Blood Adv., 2021). That steady-state metabolism within distinct circulating immune subsets is not fixed is not unexpected and further studies are required to fully define the factors that shape metabolic profiles of circulating hematopoietic cells.

Despite this apparent heterogeneity, we consistently observed that circulating ILC2 higher mitochondrial mass and polarization compared to NKDim cells (and other lymphocyte subsets including naïve T cells for example), indicating that these cells

have elevated bioenergetic properties. We include here TMRM/Mitotracker staining in blood ILC2 and NKDim cells in 12 healthy individuals. Additional 'peaks' may in fact be due to the small numbers of ILC2 that are analyzed in some samples. It is for this reason we used MFI as a comparative measure. These results are included in the revised manuscript as Extended Data Fig. 1c and discussed in the manuscript (line 96, page 4).*

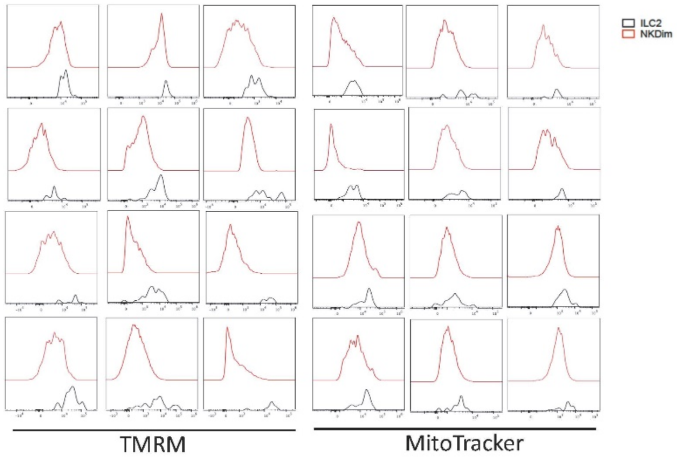

Figure 1 for reviewer
TMRM and Mitotracker staining of circulating ILC2 and NKDim cells in 12 healthy donors. Data from five independent experiments.

The overall reliance of ILC2 on polarized mitochondria is confirmed by independent data collected from patients with mitochondrial disorders and it translates into a uniform sensitivity of blood ILC2 to OXPHOS and nutrients inhibitors at steady state (see below Figure 3 for Reviewer #3), to cytokine stimulation (manuscript Figure 4) and finally to reduced oxygen exposure (manuscript Figure 3).

The authors suggest that the basis for metabolic 'uniqueness' of circulating ILC2s is differential transit through tissues or exposure to different environmental signals but do not address this experimentally. The new tonsil ILC2 data suggest that tissue residency in fact does not determine metabolic 'naivety' and comparisons with other tonsil ILC populations, particularly ILC precursors, are not shown. In addition, the lack of ST2 expression on blood/tonsil ILC2s would suggest that IL-33 does not influence metabolic changes in vivo.

We realize that our use of the term 'metabolic uniqueness' may be confusing as all cells use common and shared metabolic pathways. We were trying to emphasize the elevated metabolic activity of ILC2 compared to other innate lymphocytes (such as

NKDim cells). In the previous round of review, the reviewer requested a comparison of circulating ILC2 to other circulating subsets and to tissue ILC2. We made a comparison of blood ILC2 to blood CD4⁺ T subsets (naïve and memory) and NK cells because they have been extensively characterized from a metabolic standpoint, providing a basis to evaluate ILC2 metabolism in circulating cells. These results were previously included in the revised manuscript (Supplementary Figure 1). We have now revised the manuscript to remove the term 'metabolic uniqueness' and to instead emphasize the elevated bioenergetic properties of ILC2.

Regarding analysis of tissue ILC2, we compared blood and tonsil ILC2 from the same individuals. We found few differences between circulating ILC2 and tonsil ILC2 which may reflect the fact that we studied normal healthy tonsil samples that were not inflamed. It is difficult to experimentally study lymphocyte migration in humans and we believe that our use of paired blood/tonsil samples represents the best way to limit donor variation. Regarding interpretation, we suggested several explanations for the similar profiles of blood and tonsil ILC2. One explanation is that recovered tonsil ILC2 may include a predominant blood component. An alternative explanation is that tissue residency impacts elevated bioenergetic properties to ILC2 and that cells that return to the circulation have this 'imprint'. We have discussed these two possibilities more clearly in the revised manuscript.

The reviewer [REDACTED]

Despite the scarcity of ILC2 in tonsils, we could confirm their reliance on OXPHOS, suggesting that mitochondrial fitness remains a crucial metabolic feature of ILC2. Indeed, ILC2 do not survive hypoxic environments and have the potential to acquire flexibility in nutrients uptake upon cytokine stimulation, probably as mechanism to adapt to different environmental conditions. The lack of expression of ST2 on blood (and perhaps tissue) ILC2 is consistent with this property. As shown in previous reports (Lim A.I., JEM, 2012) and as we demonstrated in our culture system, cytokine

stimulation can upregulate ST2 expression allowing IL-33 to impact on cell metabolism.

(R4). We thank the reviewer for this comment. We analyzed effects of glycolysis inhibitors on IL-5 and amphiregulin production by cultured ILC2 (please see response to Reviewer #1, comment 3). We found that glycolysis inhibited IL-5 production (similar to that observed for IL-13). In contrast, amphiregulin production was not affected suggesting distinct metabolic regulation of ILC2-dependent production of soluble immune mediators. We have included these data Supplemental Figures 7B, C and discussed these results in the revised manuscript (lines 247-250, page 8).

Unfortunately, the authors did not evaluate IL-9, which acts in an autocrine/paracrine manner to influence ILC2 survival and fitness, as well as production of IL-5, IL-13, and amphiregulin in different settings (Wilhelm et al., Nat Immunol. 2011 Oct 9;12(11):1071-7; Turner et al., J Exp Med. 2013 Dec 16;210(13):2951-65; Mohapatra et al., Mucosal Immunology 2016 9:275–286).

We thank the reviewer for this suggestion. We have revised the introduction to include references to IL-9 in the biology of ILC2. Still, the role for IL-9 has been more extensively studied in mice (as described in the studies indicated by the reviewer), and its role in human ILC2 biology is less clear. For this reason, we focused our ILC2 metabolic analysis on well characterized cytokines (IL-5, IL-13, amphiregulin).

Reviewer #3

(Remarks to the Author)
The paper is improved by the revision, but I have a few questions based on the new emphasis on a few of the points addressed in the revision.

We thank the reviewer for the positive comments on our revised manuscript.

I have difficulty with the TMRM plot in Fig. S1F – there are multiple peaks for the ILC2s. This can also be seen in Figs. S1K and 3D. This implies that there is heterogeneity in the ILC2 population, with each peak representing a population of cells that has mitochondria with membrane potential that is distinct from the cells represented by the other peaks. I think that the authors are trying to say as much when they refer to cells having exposure to "distinct environmental signals" (page 4, line 110), but this description and related implications need to be more clearly stated in the text. Is there a precedent for this type of staining in other cell types? Have the authors attempted to gate on each of the peaks and probe for marks that discriminate between the distinct populations? This seems like an important point to me.

We thank the reviewer for these comments, which were also raised in part by Reviewer #2. As stated in our rebuttal above, mitochondrial mass and membrane heterogeneity has been reported in previous publications (Sukumar M. et al, Cell Metabolism, 2016; Callender L.A. et al, Nat Comm, 2021; Lopes N., Nat Imm, 2021) and in human innate lymphocytes (CD56+ bright and dim NK cell subsets, Surace L. et al, Blood Adv., 2021). This reflects individual variation in the human population and also the small number of gated ILC2 events analyzed in some samples. Nevertheless, the distinct bioenergetic ILC2 profile was consistently observed as shown in the following Figure for the reviewer (now included as Extended Data Fig. 1c).

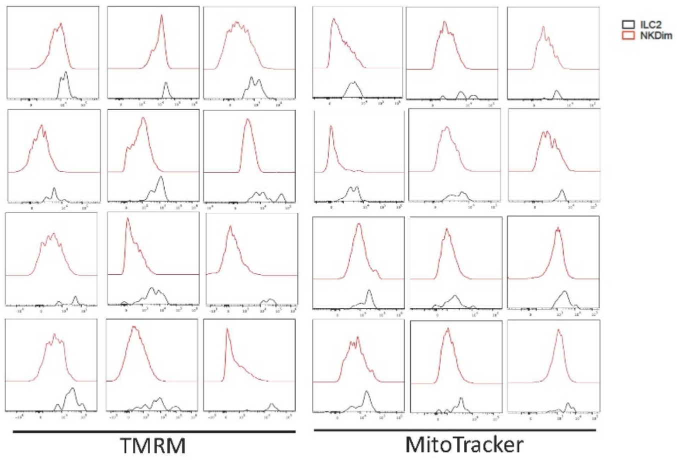

Figure 1 for reviewer
TMRM and Mitotracker staining of circulating ILC2 and NKDim cells in 12 healthy donors. Data from five independent experiments.

It is not clear to me why a similar TMRM staining pattern in tonsil pDC vs. blood pDC suggests that they are "not yet rewired metabolically by local ..signals" (line 118). Is the tissue-enforced metabolic rewiring that they are alluding to here the effects of IL-2, -7 and -33? If so, this should be explicitly stated.

We thank the reviewer for this comment, which we suppose refers to ILC2 (not pDC). As we studied healthy, non-inflamed tonsils, we suggested that the similar TMRM staining patterns might reflect that tonsil ILC2 included a predominant blood ILC2 component that had not received local signals. We have revised the text (lines 117-119, pages 4-5) to explicitly state that these signals could include IL-2, -7 and -33 or other cytokines in tissues.

It would be good to see whether any of the metabolic inhibitors that affect function result in a reduction in heterogeneity in the TMRM staining pattern - are there ILC2s , represented by peaks, that are preferentially affected by the manipulation of different metabolic pathways? I imagine that the authors have these data already, since they show data from TMRM measurements in e.g. Figs. 1F,G, 2F,G, but only as bar graphs.

The reviewer asks whether inhibitors alter the TMRM staining patterns in freshly sorted ILC2 (Fig. 1F,G and 2F,G in the revised manuscript). The data was included in the manuscript as bar graphs of MFI but we include here the staining patterns from 4 healthy donors (Figure below for the reviewer). We did not observe a heterogeneous response to the inhibitors. This result suggests that although mitochondrial polarization levels might differ in a donor-to-donor fashion, the dependance of circulating ILC2 on OXPHOS/oxygen consumption and amino acids, as building blocks to sustain mitochondrial activity, represents a fundamental property. These results have been included in the manuscript as Extended Figure 4e and commented in the manuscript (lines 163-168, page 6).

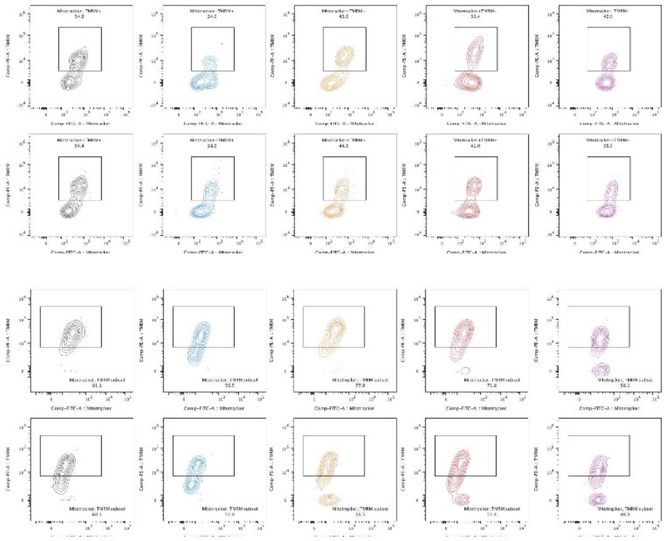

Figure 3 for reviewer
Mitotracker and TMRM incorporation were measured by FACS in freshly sorted ILC2 treated for 18h with antimycin A (blue), nor-NOHA (orange), BCAT (red) and rotenone (pink) or DMSO (black). Data from four donors from two independent experiments. Data representative of a total of three independent experiments with at least three donors each.

Page 5, line 149. LAT1 is composed of SLC3A2/CD98 and SLC7A5, and expression of the latter is not elevated, so I don't think that it is appropriate to talk about increased expression of LAT1 without functional assays or evidence of increased SCL7A5 expression. SLC3A2 also partners other SLCs in other amino acid transporters.

We thank the reviewer for this comment. We have revised the corresponding text (lines 148-150, page 5) to read:

"We detected enhanced expression of Slc3a2 (light subunit of the LAT1 receptor) and Slc43a2 (LAT4) in ILC2 (Fig. 2b). LAT1 and LAT4 preferentially transport BCAAs."

Line 170. It is not clear what the authors are referring to when they say "metabolically naïve". Please expand discussion around this idea.

We thank the reviewer for this comment. We have revised the corresponding text (lines 174-177, page 6) to read:

"Together our data show that circulating ILC2 are not in a resting metabolic state, but already show elevated profiles characterized by increased BCAA and arginine levels that sustain OXPHOS required for cell fitness and survival."

Text references to Figure 3 are muddled – the call outs in the text after 3d are incorrect.

We apologize for this confusion, we have corrected the call outs for Figure 3.

Decision Letter, second revision:

Subject: Your manuscript, NI-LE31657B

Message: Our ref: NI-LE31657B

23rd Aug 2021

Dear Dr. Di Santo,

Thank you for your patience as we've prepared the guidelines for final submission of your Nature Immunology manuscript, "Dichotomous metabolic networks govern human ILC2 proliferation and function" (NI-LE31657B). Please carefully follow the step-by-step instructions provided in the attached file, and add a response in each row of the table to indicate the changes that you have made. Please also check and comment on any additional marked-up edits we have proposed within the text. Ensuring that each point is addressed will help to ensure that your revised manuscript can be swiftly handed over to our production team.

We would like to start working on your revised paper, with all of the requested files and forms, as soon as possible (preferably within two weeks). Please get in contact with us if you anticipate delays.

When you upload your final materials, please include a point-by-point response to any remaining reviewer comments and please make sure to upload your checklist.

If you have not done so already, please alert us to any related manuscripts from your group that are under consideration or in press at other journals, or are being written up for submission to other journals (see: https://www.nature.com/nature-research/editorial-policies/plagiarism#policy-on-duplicate-publication for details).

In recognition of the time and expertise our reviewers provide to Nature Immunology's editorial process, we would like to formally acknowledge their contribution to the external peer review of your manuscript entitled "Dichotomous metabolic networks govern human ILC2 proliferation and function". For those reviewers who give their assent, we will be publishing their names alongside the published article.

Nature Immunology offers a Transparent Peer Review option for new original research manuscripts submitted after December 1st, 2019. As part of this initiative, we encourage our authors to support increased transparency into the peer review process by agreeing to have the reviewer comments, author rebuttal letters, and editorial decision letters published as a Supplementary item. When you submit your final files please clearly state in your cover letter whether or not you would like to participate in this initiative. Please note that failure to state your preference will result in delays in accepting your manuscript for publication.

Cover suggestions

As you prepare your final files we encourage you to consider whether you have any images or illustrations that may be appropriate for use on the cover of Nature

Immunology.

Covers should be both aesthetically appealing and scientifically relevant, and should be supplied at the best quality available. Due to the prominence of these images, we do not generally select images featuring faces, children, text, graphs, schematic drawings, or collages on our covers.

We accept TIFF, JPEG, PNG or PSD file formats (a layered PSD file would be ideal), and the image should be at least 300ppi resolution (preferably 600-1200 ppi), in CMYK colour mode.

If your image is selected, we may also use it on the journal website as a banner image, and may need to make artistic alterations to fit our journal style.

Please submit your suggestions, clearly labeled, along with your final files. We'll be in touch if more information is needed.

Nature Immunology has now transitioned to a unified Rights Collection system which will allow our Author Services team to quickly and easily collect the rights and permissions required to publish your work. Approximately 10 days after your paper is formally accepted, you will receive an email in providing you with a link to complete the grant of rights. If your paper is eligible for Open Access, our Author Services team will also be in touch regarding any additional information that may be required to arrange payment for your article.

You will not receive your proofs until the publishing agreement has been received through our system.

Please note that <i>Nature Immunology</i> is a Transformative Journal (TJ). Authors may publish their research with us through the traditional subscription access route or make their paper immediately open access through payment of an article-processing charge (APC). Authors will not be required to make a final decision about access to their article until it has been accepted. Find out more about Transformative Journals.

If you have any questions about costs, Open Access requirements, or our legal forms, please contact ASJournals@springernature.com.

<B>Authors may need to take specific actions to achieve compliance with funder and institutional open access mandates. For submissions from January 2021, if your research is supported by a funder that requires immediate open access (e.g. according to Plan S principles) then you should select the gold OA route, and we will direct you to the compliant route where possible. For authors selecting the subscription publication route our standard licensing terms will need to be accepted, including our self-archiving policies. Those standard licensing terms will supersede any other terms that the author or any third party may assert apply to any version of the

manuscript.

Please use the following link for uploading these materials: [REDACTED]

If you have any further questions, please feel free to contact me.

Best regards,

Elle Morris
Senior Editorial Assistant
Nature Immunology
Phone: 212 726 9207
Fax: 212 696 9752
E-mail: immunology@us.nature.com

On behalf of

Laurie A. Dempsey, Ph.D.
Senior Editor
Nature Immunology
l.dempsey@us.nature.com
ORCID: 0000-0002-3304-796X

Final Decision Letter:

Subject: Decision on Nature Immunology submission NI-LE31657C

Message: In reply please quote: NI-LE31657C

Dear Jim,

I am delighted to accept your manuscript entitled "Dichotomous metabolic networks govern human ILC2 proliferation and function" for publication in an upcoming issue of Nature Immunology.

The manuscript will now be copy-edited and prepared for the printer. Please check your calendar: if you will be unavailable to check the galley for some portion of the next month, we need the contact information of whom will be making corrections in your stead. When you receive your galleys, please examine them carefully to ensure that we have not inadvertently altered the sense of your text.

Acceptance is conditional on the data in the manuscript not being published elsewhere, or announced in the print or electronic media, until the embargo/publication date. These restrictions are not intended to deter you from presenting your data at academic meetings and conferences, but any enquiries from the media about papers not yet scheduled for publication should be referred to us.

Please note that <i>Nature Immunology</i> is a Transformative Journal (TJ). Authors may publish their research with us through the traditional subscription access route or

make their paper immediately open access through payment of an article-processing charge (APC). Authors will not be required to make a final decision about access to their article until it has been accepted. Find out more about Transformative Journals.

<B>Authors may need to take specific actions to achieve compliance with funder and institutional open access mandates. For submissions from January 2021, if your research is supported by a funder that requires immediate open access (e.g. according to Plan S principles) then you should select the gold OA route, and we will direct you to the compliant route where possible. For authors selecting the subscription publication route our standard licensing terms will need to be accepted, including our self-archiving policies. Those standard licensing terms will supersede any other terms that the author or any third party may assert apply to any version of the manuscript.

In approximately 10 business days you will receive an email with a link to choose the appropriate publishing options for your paper and our Author Services team will be in touch regarding any additional information that may be required.

You will not receive your proofs until the publishing agreement has been received through our system.

If you have any questions about our publishing options, costs, Open Access requirements, or our legal forms, please contact ASJournals@springernature.com

Once your manuscript is typeset and you have completed the appropriate grant of rights, you will receive a link to your electronic proof via email with a request to make any corrections within 48 hours. If, when you receive your proof, you cannot meet this deadline, please inform us at rjsproduction@springernature.com immediately. Once your paper has been scheduled for online publication, the Nature press office will be in touch to confirm the details.

Your paper will be published online soon after we receive your corrections and will appear in print in the next available issue. Content is published online weekly on Mondays and Thursdays, and the embargo is set at 16:00 London time (GMT)/11:00 am US Eastern time (EST) on the day of publication. Now is the time to inform your Public Relations or Press Office about your paper, as they might be interested in promoting its publication. This will allow them time to prepare an accurate and satisfactory press release. Include your manuscript tracking number (NI-LE31657C) and the name of the journal, which they will need when they contact our office.

About one week before your paper is published online, we shall be distributing a press release to news organizations worldwide, which may very well include details of your work. We are happy for your institution or funding agency to prepare its own press release, but it must mention the embargo date and Nature Immunology. Our Press Office will contact you closer to the time of publication, but if you or your Press Office have any enquiries in the meantime, please contact press@nature.com.

Also, if you have any spectacular or outstanding figures or graphics associated with your manuscript - though not necessarily included with your submission - we'd be delighted to consider them as candidates for our cover. Simply send an electronic version (accompanied by a hard copy) to us with a possible cover caption enclosed.

To assist our authors in disseminating their research to the broader community, our SharedIt initiative provides you with a unique shareable link that will allow anyone (with or without a subscription) to read the published article. Recipients of the link with a subscription will also be able to download and print the PDF.

As soon as your article is published, you will receive an automated email with your shareable link.

You can now use a single sign-on for all your accounts, view the status of all your manuscript submissions and reviews, access usage statistics for your published articles and download a record of your refereeing activity for the Nature journals.

If you have not already done so, we strongly recommend that you upload the step-by-step protocols used in this manuscript to the Protocol Exchange. Protocol Exchange is an open online resource that allows researchers to share their detailed experimental know-how. All uploaded protocols are made freely available, assigned DOIs for ease of citation and fully searchable through nature.com. Protocols can be linked to any publications in which they are used and will be linked to from your article. You can also establish a dedicated page to collect all your lab Protocols. By uploading your Protocols to Protocol Exchange, you are enabling researchers to more readily reproduce or adapt the methodology you use, as well as increasing the visibility of your protocols and papers. Upload your Protocols at www.nature.com/protocolexchange/. Further information can be found at www.nature.com/protocolexchange/about .

Please note that we encourage the authors to self-archive their manuscript (the accepted version before copy editing) in their institutional repository, and in their funders' archives, six months after publication. Nature Research recognizes the efforts of funding bodies to increase access of the research they fund, and strongly encourages authors to participate in such efforts. For information about our editorial policy, including license agreement and author copyright, please visit www.nature.com/ni/about/ed_policies/index.html

An online order form for reprints of your paper is available at https://www.nature.com/reprints/author-reprints.html. Please let your coauthors and your institutions' public affairs office know that they are also welcome to order reprints by this method.

Sincerely,

Laurie

Laurie A. Dempsey, Ph.D.
Senior Editor
Nature Immunology

l.dempsey@us.nature.com
ORCID: 0000-0002-3304-796X
